# Versatile Co‐Engineering of Immunogenic Cell Death‐Primed Dead Tumor Cell Bodies With Tumor‐Activated MnO_2_ for cGAS‐STING‐Potentiated Personalized Immunotherapy

**DOI:** 10.1002/advs.77126

**Published:** 2026-08-12

**Authors:** Bolun Xu, Xuan Lu, Yuquan Wang, Haihua Ji, Ying Zhang, Yiqun Wan, Hao Wan

**Affiliations:** ^1^ School of Chemistry and Chemical Engineering Nanchang University Nanchang P. R. China; ^2^ State Key Laboratory of Food Science and Resources Nanchang University Nanchang P. R. China

**Keywords:** cGAS‐STING pathway activation, dead tumor cell bodies, immunogenic cell death, personalized cancer immunotherapy, tumor microenvironment responsiveness

## Abstract

Immunogenic cell death (ICD)‐based whole‐cell personalized cancer immunotherapies present full tumor antigens but suffer from poor immunogenicity, early clearance, and a glutathione (GSH)‐rich immunosuppressive tumor microenvironment (TME). Here, we report a TME‐responsive immunotherapeutic platform, DTBEI@CS@MnO_2_, which integrates immunogenicity‐enhanced dead tumor cell bodies (DTBEI) with Mn^2^^+^‐potentiated innate immunity. Tumor cells preloaded with indocyanine green (ICG) are sequentially functionalized with a chitosan (CS) interlayer followed by MnO_2_ adsorption (CS@MnO_2_) via electrostatic interactions. Upon near‐infrared laser irradiation, ICG‐mediated photodynamic/photothermal effect induces potent ICD, generating DTBEI. Within the acidic and GSH‐enriched TME, the CS@MnO_2_ coating disassembles to release DTBEI and MnO_2_. DTBEI provides endogenous antigens and ICD‐associated danger signals, while MnO_2_ is reduced by GSH to Mn^2^^+^. The resulting Mn^2^^+^ amplifies DTBEI‐ and tumor‐derived deoxyribonucleic acid‐triggered cyclic GMP‐AMP synthase (cGAS)‐stimulator of interferon genes (STING) activation and downstream immune‐stimulatory effects, thereby synergistically promoting dendritic cell maturation, antigen cross‐presentation, and tumor‐specific T‐cell priming. Consequently, DTBEI@CS@MnO_2_ elicits durable anti‐tumor immunity, effectively inhibiting tumor growth and suppressing recurrence and metastasis, while establishing T‐cell memory responses. Importantly, this strategy shows broad applicability in multiple tumor models, including CT26 colon cancer, 4T1 breast cancer, and B16F10 melanoma, while maintaining favorable biocompatibility. Overall, these findings highlight a materials‐based approach for personalized cancer immunotherapy.

## Graphical abstract

DTBEI@CS@MnO_2_, fabricated via modular electrostatic co‐engineering and photothermal/photodynamic immunogenic cell death, specifically responds to the acidic and GSH‐rich tumor microenvironment. This triggers the release of DTBEI that provides tumor antigens, DNA, and DAMPs, as well as the generation of Mn^2^^+^, thereby synergistically potentiating cGAS‐STING signaling and eliciting robust anti‐tumor immunity for personalized cancer therapy.

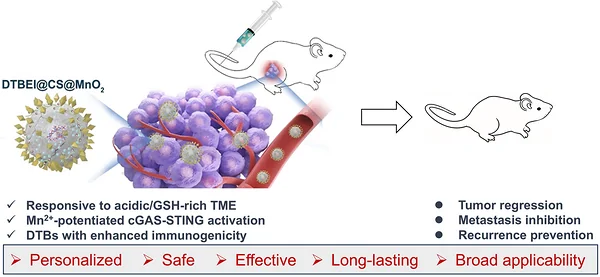

## 1 Introduction

Cancer immunotherapy represents a major breakthrough in modern oncology, driving continuous exploration of personalized therapeutic strategies that can elicit durable and systemic anti‐tumor immune responses [1, 2, 3]. In this regard, whole‐cell therapeutic platforms derived from dead tumor cell bodies (DTBs) are particularly attractive, as they preserve the full spectrum of clonal and subclonal tumor antigens, thereby providing a comprehensive antigenic repertoire for immune recognition [4]. DTBs also offer a safety‐related advantage in that their non‐proliferative nature reduces concerns about tumorigenicity [5]. Nevertheless, their clinical translation remains limited by the insufficient immunogenic amplification and poor in vivo delivery stability of DTB‐based therapeutics, which necessitates engineering strategies to enable efficient immune activation.

Immunogenic cell death (ICD) represents a biologically based strategy for addressing this challenge by promoting the release or increased availability of tumor antigens together with the exposure or release of danger‐associated molecular patterns (DAMPs) [6, 7, 8]. These ICD‐related signals facilitate dendritic cell (DC) maturation and antigen cross‐presentation, thereby initiating tumor‐specific T‐cell responses. Importantly, under laser irradiation, photosensitizers and photothermal agents can effectively induce ICD through the generation of reactive oxygen species (ROS) and heat, respectively [9, 10, 11, 12]. This process generates DTBs with enhanced immunogenicity (DTBEI), which preserve a broad spectrum of endogenous tumor antigens and simultaneously present ICD‐associated danger signals. From a biomaterial perspective, DTBEI can be considered multifunctional immunogenic components that can be engineered at the surface for stabilization, delivery, and immune enhancement. Nevertheless, the therapeutic efficacy of ICD‐derived whole‐cell immunotherapeutics is still limited within the immunosuppressive tumor microenvironment (TME). In particular, the reductive nature of the TME restrains innate immune sensing and makes it difficult to sustain effective adaptive immune priming.

Material‐based encapsulation provides a promising strategy for stabilizing and delivering DTBEI. However, conventional chemical encapsulation strategies often involve harsh processing conditions that may compromise antigen integrity and disrupt immunogenic signals [13, 14]. In comparison, biocompatible surface adsorption provides a mild and non‐damaging approach for constructing protective layers around biological substrates. Chitosan (CS), a biocompatible cationic polysaccharide, is particularly attractive in this context due to its strong electrostatic affinity for negatively charged cellular components, thereby enabling the formation of stable yet flexible coatings [15, 16, 17].

Although CS‐based encapsulation can improve the stability and delivery of DTBs, effective cancer immunotherapy also requires active remodeling of the immunosuppressive TME to enhance immune priming and effector function. In the TME, elevated glutathione (GSH) levels help maintain a reductive microenvironment that lowers oxidative stress, suppresses innate immune activation, and limits antigen‐driven adaptive immune responses [18, 19, 20]. In this regard, manganese dioxide (MnO_2_) is regarded as a TME‐responsive inorganic material. By selectively reacting with GSH through redox processes, MnO_2_ not only disrupts tumor redox homeostasis but also generates Mn^2^^+^ ions, which serve as a potent cofactor for innate immune sensing, particularly by amplifying deoxyribonucleic acid (DNA)‐triggered cyclic GMP‐AMP synthase (cGAS)‐stimulator of interferon genes (STING) signaling [21, 22, 23].

Guided by these considerations, we developed a co‐engineering strategy that combined DTBEI with materials‐enabled modulation of the TME. In this design, DTBEI were modified with a predominantly surface‐associated CS interlayer and GSH‐responsive MnO_2_ adsorbates (CS@MnO_2_) through sequential electrostatic assembly. This platform brought together antigen preservation, immunogenic enhancement, and innate immune pathway potentiation. Under the combined action of photodynamic and photothermal effects, tumor cells underwent ICD, thereby generating DTBEI. Upon reaching the TME, the acidic environment promoted the degradation of CS@MnO_2_, while GSH reduced Mn^4^^+^ to Mn^2^^+^ simultaneously consumed GSH, and remodeled the redox state of the TME. The released Mn^2^^+^ ions associated with DNA from DTBEI as well as tumor cells, forming an “immune activation complex” that activates the cGAS‐STING signaling pathway. Together with DTBEI, this activation promoted dendritic cell (DC) maturation, cytokine secretion, and tumor‐specific T cell activation, ultimately leading to enhanced anti‐tumor immune responses and long‐lasting immune memory (Figure 1). The efficacy and general applicability of this strategy were demonstrated in different tumor models, including CT26 colon cancer, 4T1 breast cancer, and B16F10 melanoma. Collectively, this strategy presents a promising and translatable approach for personalized cancer immunotherapy, providing new avenues for effective cancer treatment.

**FIGURE 1:**
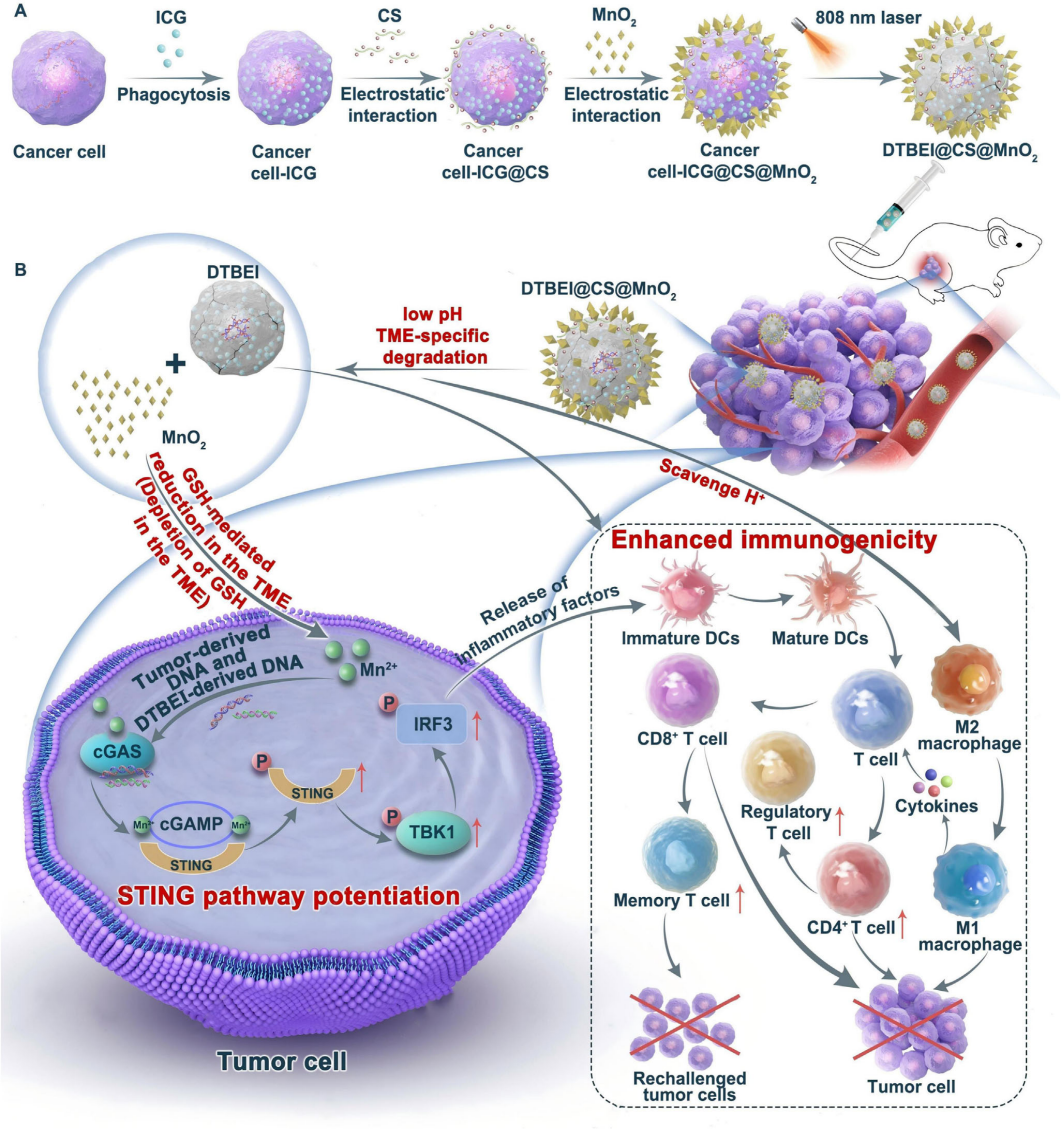
Schematic illustration of the design and mechanism of DTBEI@CS@MnO_2_ for TME‐responsive personalized cancer immunotherapy. (A) Synthesis of DTBEI@CS@MnO_2_: cancer cells were preloaded with ICG, followed by electrostatic assembly with CS to form cancer cell‐ICG@CS. Anionic MnO_2_ was subsequently adsorbed onto the CS layer, resulting in the formation of cancer cell‐ICG@CS@MnO_2_. Upon 808 nm laser irradiation, the photothermal/photodynamic effects of ICG induce ICD, converting the cells into DTBEI surrounded by a predominantly surface‐associated CS@MnO_2_ coating. (B) In vivo tumor‐specific immune activation mechanism: After intravenous injection, DTBEI@CS@MnO_2_ reached the TME, where the acidic and GSH‐enriched environment triggered the disassembly of CS@MnO_2_, accompanied by the sequential release of DTBEI and MnO_2_. Intratumoral GSH subsequently reduced Mn^4^^+^ to Mn^2^^+^; the released Mn^2^^+^ then formed an “immune activation complex” with tumor‐ and DTBEI‐derived DNA, activating the cGAS‐STING pathway. This activation, along with DTBEI, synergistically promoted DC maturation and stimulated CD8^+^ cytotoxic T lymphocyte (CTL) and CD4^+^ T cell responses. The immune cascade resulted in the generation of memory T cells, reprogramming of the immunosuppressive TME, and secretion of immunomodulatory cytokines. Ultimately, these effects inhibited tumor growth and reduced the risks of both recurrence and metastasis.

## 2 Results and Discussion

### 2.1 Synthesis and Characterization of DTBEI@CS@MnO_2_

To establish an immunogenic tumor‐derived antigen source, CT26 cells were first loaded with ICG to obtain CT26‐ICG. ICG is a clinically approved photosensitizer capable of generating ROS and heat under 808 nm laser irradiation [24, 25]. After 8 min of laser exposure, CT26‐ICG exhibited pronounced photodynamic and photothermal effects (Figures S1 and S2), leading to extensive tumor‐cell death and DTBEI generation (Figure S3). To further verify that DTBEI generation was accompanied by ICD‐associated danger signals, representative DAMP markers were examined under the same cell‐equivalent conditions. CT26 cells, CT26 cells treated with 808 nm laser alone, and CT26‐ICG cells without laser irradiation were used as negative controls, while doxorubicin (DOX)‐treated CT26 cells were included as a positive ICD control. Compared with the negative‐control groups, CT26‐ICG cells after 808 nm laser irradiation (i.e., DTBEI) showed markedly increased extracellular adenosine triphosphate (ATP) secretion (Figure S4), calreticulin (CRT)‐associated extracellular signal (Figure S5), and high mobility group box 1 protein (HMGB1) release (Figure S6). The ATP and CRT‐associated levels in DTBEI were comparable to those in the DOX‐positive control group. HMGB1 release was lower than that induced by DOX but remained significantly higher than that in all negative‐control groups. These results indicate that 808 nm laser irradiation of CT26‐ICG cells generated DTBEI with a clear ICD‐associated DAMP profile. Flow cytometry analyses further demonstrated that DTBEI exhibited superior immunogenicity compared to commercially available buffer‐induced inactivation (LBTB). This advantage was supported by key indicators, including robust DC maturation (Figure S7) and significant activation of CD3^+^CD8^+^ (Figure S8) as well as CD3^+^CD4^+^ T cells (Figure S9). Accordingly, DTBEI were adopted as the solid antigen source for subsequent immunotherapy investigations. Concurrently, CS functioned both as a protective surface layer for DTBEI and as an interfacial adsorption medium for MnO_2_, resulting in the formation of DTBEI@CS@MnO_2_. The CS‐mediated electrostatic adsorption strategy was chosen instead of in situ biomimetic mineralization because it provides a milder and more controllable interfacial assembly process [15, 16, 17]. In situ mineralization usually involves metal precursor exposure and local nucleation/growth reactions, which may irreversibly perturb tumor antigens or ICD‐associated signals on DTBEI [26, 27]. By contrast, electrostatic adsorption enables the stepwise construction of a CS@MnO_2_ surface coating under mild conditions. Although the resulting coating may transiently reduce the extracellular accessibility of surface‐associated immunogenic signals through steric shielding, it preserves their integrity and allows their accessibility to be restored following TME‐responsive coating disassembly.

Briefly, CT26‐ICG was incubated with CS to form CT26‐ICG@CS via electrostatic interaction between the positively charged CS and negatively charged CT26‐ICG cell membranes. During this process, zeta‐potential profiling indicated that 200 µg/mL CS and a 15‐min incubation were the lowest concentration–shortest time pair that fully reversed the cell surface charge (Figures S10 and S11); this condition was therefore adopted to generate CT26‐ICG@CS. To further verify the microscopic localization of the CS layer, cyanine 3 (Cy3)‐labeled CS and DiO membrane staining were used for confocal laser scanning microscopy (CLSM) observation under the same assembly conditions. Cy3‐CS showed a ring‐like signal that largely overlapped with the DiO‐labeled plasma membrane. Z‐stack images confirmed that the signal was mainly distributed at the cell periphery (Figure S12). These results indicate that CS formed a predominantly surface‐associated coating on CT26‐ICG cells. Subsequently, MnO_2_ was introduced and incubated for 40 min to form CT26‐ICG@CS@MnO_2_ (Figure S13). Finally, CT26‐ICG@CS@MnO_2_ was irradiated with an 808 nm laser at 1.0 W/cm^2^ for 8 min, inducing ICD in the inner CT26‐ICG cells and generating DTBEI surrounded by a predominantly surface‐associated CS@MnO_2_ coating. The resulting formulation was denoted as DTBEI@CS@MnO_2_. In contrast, free DTBEI was prepared by directly irradiating CT26‐ICG cells without CS@MnO_2_ coating and was used as a control. To further quantify the loading amounts of CS, a parallel DTBEI@CS‐FITC@MnO_2_ formulation was prepared using fluorescein‐5‐isothiocyanate (FITC)‐labeled CS under the same assembly conditions. Based on the fluorescence calibration curve of FITC‐labeled CS (Figures S14 and S15), the CS loading efficiency was calculated to be 58.7%, corresponding to a CS loading amount of 117.4 µg/mL. In addition, the Mn content in the purified DTBEI@CS@MnO_2_ formulation was determined by inductively coupled plasma mass spectrometry (ICP‐MS) after acid digestion. The MnO_2_‐equivalent loading amount was calculated to be 0.123 mg/mL, and the MnO_2_ loading efficiency was 28.3% based on the initial MnO_2_ feeding concentration of 0.435 mg/mL.

Scanning electron microscopy (SEM) and transmission electron microscopy (TEM) images (Figure 2A) visually validated the coating effect: compared to the spiky surface of native uncoated CT26 cells, CT26‐ICG@CS@MnO_2_ exhibited a more compact and uniform surface appearance. Subsequently, zeta potential analysis (Figure 2B) showed a marked shift following the introduction of the outer layer, further supporting the successful assembly of the platform. As shown by energy‐dispersive x‐ray spectroscopy (EDS) analysis (Figure 2C and Figure S16), DTBEI@CS@MnO_2_ was also successfully prepared. Its elemental profile showed Mn derived from MnO_2_ as a major component, along with characteristic DTBEI elements such as P and S. Consistently, EDS line‐scan analysis also showed a significant increase in Mn intensity when the scanning path entered the material region marked in the corresponding SEM image (Figure S17). More detailed chemical information was obtained from x‐ray photoelectron spectroscopy (XPS; Figure 2D). XPS analysis was first performed on pure MnO_2_ to verify its characteristic chemical state. The high‐resolution Mn 2p spectra of MnO_2_ displayed two peaks at approximately 642.2 and 654.1 eV, corresponding to Mn 2p_3_/_2_ and Mn 2p_1_/_2_ [22], respectively, consistent with MnO_2_ composition. Subsequently, XPS of DTBEI@CS@MnO_2_ also exhibited clear Mn 2p_3_/_2_ and Mn 2p_1_/_2_ signals at similar positions, confirming the presence of Mn‐containing species in the final formulation. In addition, the stability of DTBEI@CS@MnO_2_ and uncoated CT26‐ICG was compared in ultrapure water over a period of 4 h. DTBEI@CS@MnO_2_ maintained an intact morphology. In contrast, CT26‐ICG showed clear structural disruption, likely because it lacked the protective CS@MnO_2_ coating (Figure 2E). This result emphasizes that CS@MnO_2_ encapsulation plays a key role in maintaining the structural integrity of DTBEI, thereby providing the basis for its functional performance within the TME. In addition, the incorporation of ICG enabled DTBEI@CS@MnO_2_ to emit fluorescence under 808 nm laser irradiation (Figure 2F). This fluorescence allowed its in vivo distribution to be tracked. Furthermore, DTBEI@CS@MnO_2_ demonstrated improved stability in phosphate‐buffered saline (PBS). Over a one‐week period, its zeta potential showed negligible variation, and no visible aggregation was observed (Figure 2G and Figure S18), indicating its suitability for further biological applications.

**FIGURE 2:**
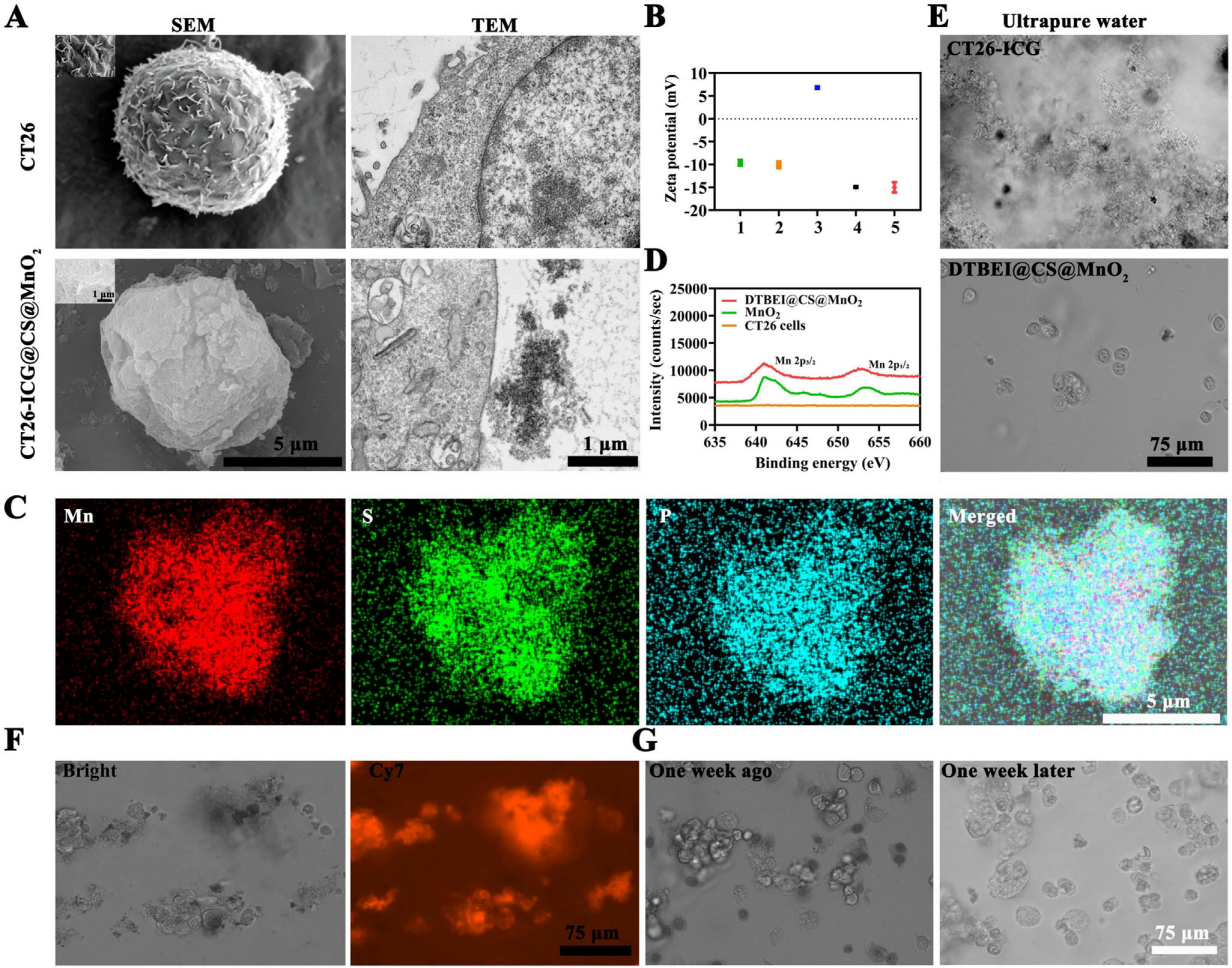
Characterization of DTBEI@CS@MnO_2_. (A) SEM and TEM images comparing CT26 cells and CT26‐ICG@CS@MnO_2_, with scale bars of 5 µm (low‐magnification SEM) and 1 µm (high‐magnification SEM and TEM). (B) Zeta‐potential measurements of sequential intermediates in the fabrication process of DTBEI@CS@MnO_2_ (*n* = 3): 1) CT26 cells; 2) CT26‐ICG; 3) CT26‐ICG@CS; 4) CT26‐ICG@CS@MnO_2_; and 5) DTBEI@CS@MnO_2_. (C) Elemental mapping analysis of DTBEI@CS@MnO_2_. Scale bar, 5 µm. (D) XPS spectra confirming the presence of Mn in DTBEI@CS@MnO_2_. (E) Micrographs illustrating CT26‐ICG and DTBEI@CS@MnO_2_ after 4 h exposure to ultrapure water. Scale bar, 75 µm. (F) Bright‐field (left) and cyanine‐7 (Cy7) channel fluorescence imaging (right) of DTBEI@CS@MnO_2_. Cy7 channel matches the fluorescence emission spectrum of ICG. Scale bar, 75 µm. (G) Micrographs of DTBEI@CS@MnO_2_ maintained in PBS for seven days. Scale bar, 75 µm. Data are expressed as mean ± standard deviation (SD).

To further evaluate whether the CS@MnO_2_ coating could protect ICD‐associated DAMPs from premature loss, HMGB1 retention behavior was examined using different DTBEI‐based formulations. Under physiological pH 7.4 conditions, free DTBEI showed a high HMGB1 leakage ratio immediately after incubation, indicating poor retention of HMGB1 in uncoated DTBEI. DTBEI@CS and DTBEI@CS@MnO_2_ exhibited low HMGB1 leakage under pH 7.4 conditions, confirming that the CS coating effectively prevented premature HMGB1 loss. In addition, CT26‐ICG@CS@MnO_2_ without laser irradiation showed only background‐level HMGB1 release, indicating that the coating process itself did not cause obvious DAMP leakage before ICD induction. By contrast, DTBEI@CS@MnO_2_ showed pronounced HMGB1 release under pH 6.5 plus GSH conditions, suggesting that the coating could preserve HMGB1 under physiological conditions while allowing TME‐triggered DAMP release (Figure S19).

We next examined whether the CS‐containing coating affected the extracellular accessibility of CRT, a representative ICD‐associated surface DAMP. Untreated CT26 cells and CT26‐ICG cells without laser irradiation showed only background CRT‐positive populations of 0.36% and 0.31%, respectively. In contrast, free DTBEI exhibited a markedly increased CRT‐positive population of 98.70%, confirming strong ICD‐associated CRT exposure following laser irradiation. After CS encapsulation, the detectable CRT‐positive population decreased to 0.28%. Similarly, DTBEI@CS@MnO_2_ maintained under pH 7.4 conditions showed only 0.34% CRT‐positive events, indicating that the CS‐containing coating markedly restricted antibody access to surface CRT under physiological conditions. Importantly, after incubation under pH 6.5 plus GSH conditions, the CRT‐positive population increased to 98.80%, reaching a level comparable to that of free DTBEI (Figure S20). These findings suggest that CS‐based encapsulation transiently masks antibody‐accessible CRT under physiological conditions, whereas acidic and reductive conditions restore CRT accessibility, consistent with TME‐responsive coating disassembly.

Following this, we continued to investigate whether it also attenuated broader storage‐associated protein loss. Freshly prepared DTBEI and DTBEI@CS@MnO_2_ were included as matched day‐0 baseline controls. Label‐free quantitative proteomic analysis was first performed to examine membrane‐associated proteins before and after storage at 37°C for 7 days. Rank‐abundance analysis revealed a broad reduction in membrane‐associated protein abundance in day‐7 free DTBEI. In contrast, day‐7 DTBEI@CS@MnO_2_ exhibited consistently higher normalized abundance across the ranked membrane‐associated protein set (Figure S21). Heatmap analysis further illustrated the relative abundance patterns of selected representative membrane and cell‐surface proteins across the four groups. Several proteins, including the MHC class I‐associated proteins H2‐D1 (H2‐D1) and H2‐K1 (H2‐K1), cluster of differentiation 44 (CD44), integrin β1 (Itgb1), integrin α5 (Itga5), E‐cadherin (Cdh1), and cluster of differentiation 47 (CD47), showed relatively lower abundance in day‐7 free DTBEI and comparatively higher abundance in day‐7 DTBEI@CS@MnO_2_ (Figure S22). These proteomic results indicate that prolonged storage caused a broad reduction in membrane‐associated protein abundance in free DTBEI, whereas this reduction was less pronounced in DTBEI@CS@MnO_2_. Time‐course sodium dodecyl sulfate‐polyacrylamide gel electrophoresis (SDS‐PAGE) analysis was subsequently performed using equal tumor‐cell‐equivalent loading. Free DTBEI showed a progressive decrease in recoverable total‐protein signal after storage at 37°C for 1, 3, and 7 days. In comparison, DTBEI@CS@MnO_2_ maintained substantially higher total‐lane intensities throughout the same storage period (Figures S23 and S24). These findings demonstrate a time‐dependent loss of recoverable proteins in free DTBEI and show that this loss was attenuated by the CS@MnO_2_ coating. Western blot (WB) analysis further showed marked reductions in HMGB1, heat shock protein 90 (HSP90), epithelial cell adhesion molecule (EpCAM), and integrin β1 in free DTBEI after 7 days of storage. In comparison, these proteins were comparatively better preserved in day‐7 DTBEI@CS@MnO_2_. HMGB1, HSP90, and integrin β1 remained significantly more abundant in day‐7 DTBEI@CS@MnO_2_ than in day‐7 free DTBEI. EpCAM was markedly reduced in day‐7 free DTBEI, whereas no statistically significant difference was observed between fresh DTBEI@CS@MnO_2_ and day‐7 DTBEI@CS@MnO_2_ under the tested conditions (Figures S25 and S26). Collectively, the proteomic, SDS‐PAGE, and WB results demonstrate that free DTBEI undergoes a progressive storage‐associated loss of recoverable proteins together with a broad reduction in membrane‐associated protein abundance. The CS@MnO_2_ coating attenuated these changes and improved the preservation of representative intracellular immunogenic and membrane‐associated proteins.

### 2.2 Dual pH/GSH‐Triggered DTBEI@CS@MnO_2_ Enables Tumor‐ Microenvironment‐Responsive Immune Activation

Building on the above physicochemical characterization, we next investigated whether the pH/GSH‐responsive disassembly of DTBEI@CS@MnO_2_ could be translated into a tumor‐specific immune activation cascade. To clarify the individual and combined effects of acidic pH and GSH on DTBEI@CS@MnO_2_ disassembly, we performed a 2 × 2 orthogonal release experiment using pH and GSH as independent variables. DTBEI release was examined under four conditions: pH 7.4 without GSH, pH 6.5 without GSH, pH 7.4 with 1 mm GSH, and pH 6.5 with 1 mm GSH. DTBEI@CS@MnO_2_ showed limited release under pH 7.4 without GSH, indicating good stability under physiological‐like conditions. Acidic pH alone moderately enhanced DTBEI release, suggesting destabilization of the CS‐containing coating. GSH alone also promoted partial disassembly, consistent with GSH‐mediated MnO_2_ reduction. The most pronounced and sustained DTBEI release was observed when acidic pH and GSH were present together (Figure 3A). These results indicate that acidic pH and GSH facilitate the disassembly of DTBEI@CS@MnO_2_ under TME‐mimicking conditions. We further assessed the redox‐responsive behavior of DTBEI@CS@MnO_2_ using a methylene blue (MB) bleaching assay [28]. The results showed that a pronounced reduction in the 665 nm absorbance peak of oxidized MB was observed after co‐incubation with H_2_O_2_, GSH, and DTBEI@CS@MnO_2_, while other controls showed little change (Figure 3B), demonstrating substantial redox responsiveness in a tumor‐mimicking environment. After confirming the tumor‐mimicking activation behavior, we next investigated its downstream immunological effects. To ensure a fair comparison, DTBEI@CS@MnO_2_ was assessed alongside MnO_2_ alone, free DTBEI, DTBEI@CS, and the physical mixture (DTBEI+CS+MnO_2_), with the DTBEI amount and corresponding component concentrations kept consistent among the relevant groups. After incubation under pH 6.5 or pH 7.4 conditions, the resulting samples were applied to bone marrow‐derived dendritic cells (BMDCs) for DC maturation analysis and to splenocytes for downstream T‐cell and cytokine evaluation. Compared with the pH 7.4‐conditioned samples, pH 6.5‐conditioned DTBEI@CS@MnO_2_ induced stronger immune activation, consistent with its acid‐responsive release behavior. In particular, pH 6.5‐conditioned DTBEI@CS@MnO_2_ markedly promoted BMDC maturation, with the proportion of CD80^+^CD86^+^ cells reaching 19.50%. In contrast, the pH 7.4‐conditioned formulation induced only weak BMDC maturation (Figure 3C), probably due to insufficient DTBEI release under neutral conditions. Consistent with the enhanced maturation of antigen‐presenting cells, pH 6.5‐conditioned DTBEI@CS@MnO_2_ also increased the proportions of CD3^+^CD8^+^ and CD3^+^CD4^+^ T‐cell populations in splenocytes to 12.00% and 17.40%, respectively (Figure 3D,E). In addition, the levels of interferon‐gamma (IFN‐γ), interleukin‐6 (IL‐6), tumor necrosis factor‐alpha (TNF‐α), and interleukin‐12 (IL‐12) in the culture supernatants were markedly increased after treatment with pH 6.5‐conditioned DTBEI@CS@MnO_2_ (Figure 3F–I). In addition to secreted cytokines, total IFN‐γ levels in splenocyte lysates were also quantified. Consistently, splenocytes treated with pH 6.5‐conditioned DTBEI@CS@MnO_2_ showed elevated intracellular IFN‐γ levels compared with the corresponding control groups (Figure S27), further supporting enhanced splenic immune activation. Notably, DTBEI@CS induced an immune response comparable to that of free DTBEI, indicating that the CS coating did not impair the intrinsic immunogenicity of DTBEI. However, DTBEI@CS@MnO_2_ produced stronger BMDC maturation, higher CD3^+^CD4^+^ and CD3^+^CD8^+^ T‐cell proportions, increased cytokine secretion, and elevated total IFN‐γ levels in splenocyte lysates compared with DTBEI@CS under pH 6.5 conditions. These results suggest that MnO_2_ provided additional immune‐potentiating effects beyond the CS coating. Moreover, DTBEI@CS@MnO_2_ was more effective than the physical mixture DTBEI+CS+MnO_2_, indicating that the integrated CS@MnO_2_ coating was important for achieving efficient immune activation under tumor‐mimicking acidic conditions. Collectively, these findings demonstrate that DTBEI@CS@MnO_2_ can convert tumor‐associated pH/GSH characteristics into localized immune enhancement, thereby enabling focused and tumor‐restricted immune activation.

**FIGURE 3:**
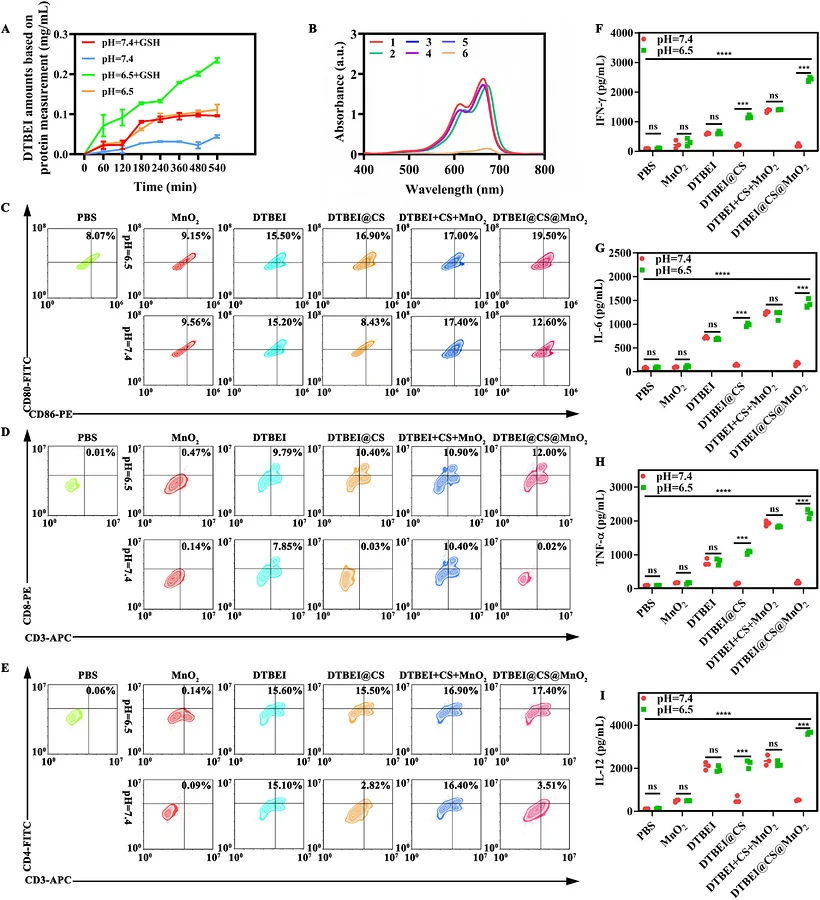
DTBEI@CS@MnO_2_ induced efficient tumor‐specific immune responses in vitro. (A) Orthogonal evaluation of pH/GSH‐triggered DTBEI release from DTBEI@CS@MnO_2_. Cumulative release profiles of DTBEI from DTBEI@CS@MnO_2_ were examined in PBS under four orthogonal conditions: pH 7.4 without GSH, pH 6.5 without GSH, pH 7.4 with 1 mm GSH, and pH 6.5 with 1 mm GSH (*n* = 3). (B) Redox‐responsive behavior of DTBEI@CS@MnO_2_ evaluated by MB bleaching assay. MB bleaching was monitored by measuring the absorbance at 665 nm after incubation under different conditions: 1: MB, 2: MB+H_2_O_2_, 3: MB+GSH, 4: MB+H_2_O_2_+DTBEI@CS@MnO_2_, 5: MB+GSH+DTBEI@CS@MnO_2_, and 6: MB+H_2_O_2_+GSH+DTBEI@CS@MnO_2_. (C) Flow cytometry evaluation of BMDC maturation markers CD86/CD80. The proportion of mature BMDCs was analyzed following treatment with different substances collected from buffer solutions (pH 6.5 or 7.4) that had been incubated with various formulations (i.e., MnO_2_, DTBEI, DTBEI@CS, DTBEI+CS+MnO_2_, and DTBEI@CS@MnO_2_). (D, E) Flow cytometry analysis of T cell subsets in splenocytes, treated with samples collected from pH 6.5 or 7.4 buffer solutions pre‐incubated with various formulations: (D) The CD3^+^CD8^+^ T cell subset (Q2 quadrant, representing the percentage of CD3^+^CD8^+^ T cells); (E) The CD3^+^CD4^+^ T cell subset (Q2 quadrant, indicating the percentage of CD3^+^CD4^+^ T cells). (F–I) Corresponding secretion levels of cytokines (i.e., IFN‐γ, IL‐6, TNF‐α, and IL‐12; *n* = 3). Data are expressed as mean ± SD. Statistical comparisons were conducted via one‐way Analysis of Variance (ANOVA) followed by Tukey's multiple comparisons test. Significance was set at ^*^ *p* < 0.05 and ^****^ *p* < 0.0001; “ns” denotes non‐significant differences.

### 2.3 In Vivo Therapeutic Efficacy of DTBEI@CS@MnO_2_ in Subcutaneous and Orthotopic CT26 Tumor Models

Based on the potent in vitro immunogenicity profile, we next evaluated the in vivo therapeutic performance of DTBEI@CS@MnO_2_ in CT26 tumor‐bearing mice. To preserve tumor homology, the formulation was prepared from CT26‐derived DTBEI and tested in syngeneic CT26 tumor‐bearing mice. Real‐time ICG fluorescence imaging showed an enhanced tumor‐localized signal after intravenous administration, reaching a maximum at 10 h post‐injection (Figure 4A). However, because ICG is a small‐molecule fluorophore that may dissociate from cell bodies in vivo, this signal was regarded as preliminary ICG‐associated tracking rather than direct evidence of intact DTBEI@CS@MnO_2_ accumulation. To further evaluate the membrane/cell‐body‐associated biodistribution of DTBEI@CS@MnO_2_, additional tracking experiments were performed using DiD, a lipophilic membrane‐associated fluorescent dye [29]. DiD‐labeled DTBEI@CS@MnO_2_ showed relatively good labeling stability in fetal bovine serum (FBS)‐containing medium over 72 h (Figure S28). In vivo imaging after intravenous injection showed that DiD‐labeled DTBEI@CS@MnO_2_ produced a more sustained tumor‐associated fluorescence signal than free DiD (Figure S29). Ex vivo fluorescence imaging of tumors, major organs, and lymph nodes collected at 10 h post‐injection further showed detectable DiD signals in tumors, lymph nodes, liver, spleen, lung, and kidney (Figure S30). These findings suggest that the tumor‐associated fluorescence signal was not solely derived from leaked free dye, but was at least partially associated with membrane/cell‐body‐related DTBEI@CS@MnO_2_ distribution. The observed tumor‐associated signal may result from multiple coordinated factors, including improved circulation stability provided by the CS@MnO_2_ coating, partial tumor‐associated vascular/perivascular retention or deposition, and potential homologous interactions between tumor‐derived cellular components and the parental tumor tissue. A preliminary dose screening suggested that DTBEI@CS@MnO_2_ containing DTBEI (1 × 10^6^ tumor‐cell equivalents) achieved the most consistent tumor growth inhibition (Figure 4B). Moreover, optimization of the dosing schedule revealed that near‐complete tumor regression was achieved only with four injections over 12 days, whereas fewer injections resulted in partial tumor growth delay (Figure 4C and Figures S31 and S32). Therefore, the four‐dose regimen of DTBEI@CS@MnO_2_ (1 × 10^6^ tumor‐cell equivalents of DTBEI per dose) was selected for subsequent therapeutic studies.

**FIGURE 4:**
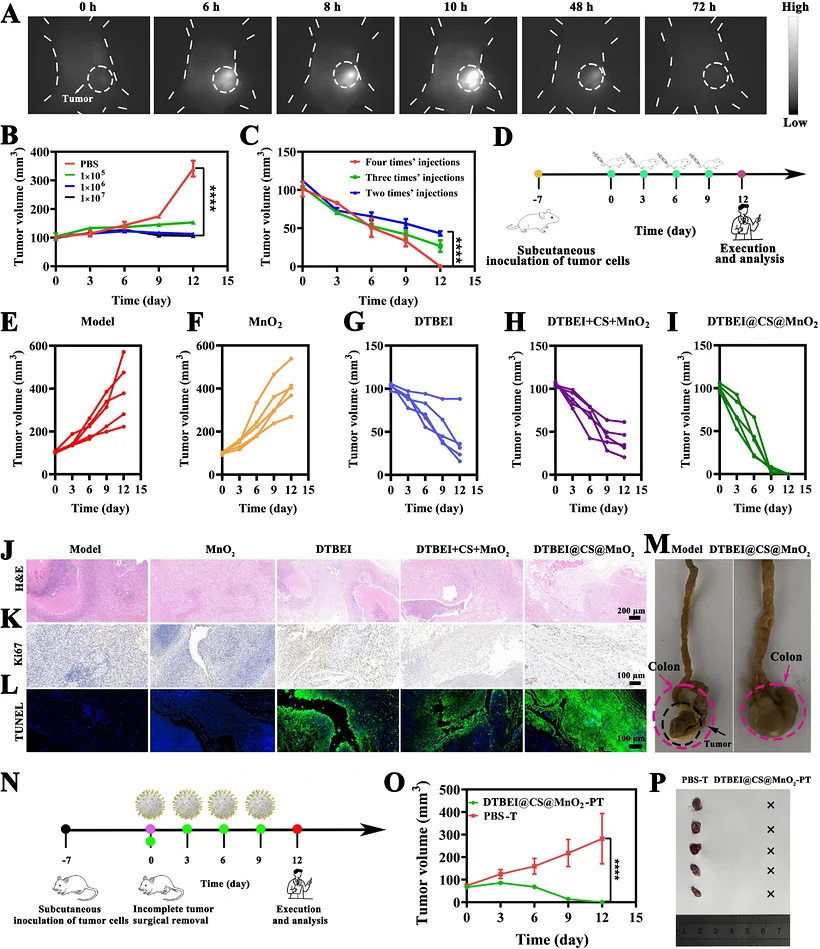
DTBEI@CS@MnO_2_ enabled robust anti‐tumor efficacy in established CT26 subcutaneous and orthotopic models and suppressed postoperative recurrence. (A) In vivo fluorescence tracking of DTBEI@CS@MnO_2_ in CT26 tumor‐bearing mice; Images were captured at 0, 6, 8, 10, 48, and 72 h post‐injection. The white dashed circle highlights the tumor. (B) Dosage optimization of DTBEI in DTBEI@CS@MnO_2_. To determine the optimal DTBEI dose, mice were given a single injection of DTBEI@CS@MnO_2_ with DTBEI quantities matching those in 1 × 10^5^, 1 × 10^6^, or 1 × 10^7^ CT26 cells, and tumor volume changes were dynamically monitored over the experimental period. (C) Tumor volume changes in mice as a function of time after DTBEI@CS@MnO_2_ injections at varying frequencies. (D) Different formulations‐based tumor treatment schematic. (E–I) Comparative analysis of CT26 tumor volume changes in mice over time across different groups. (J–L) H&E, Ki67, and TUNEL staining of tumors collected from different groups. Scale bars, 200, 100, and 100 µm, respectively. (M) Digital images of colons from orthotopic colon tumor mice treated with PBS or DTBEI@CS@MnO_2_. The black dashed circle highlights the tumor and the red dashed circle indicates the colon. (N) Schematic illustration of personalized postoperative treatment based on DTBEI@CS@MnO_2_. (O) Tumor volume growth curves in mice after receiving personalized postoperative treatment (*n* = 5). P) Resected tumors at the endpoint after personalized postoperative treatment (*n* = 5). Data are expressed as mean ± SD. One‐way ANOVA was used for statistical analysis, with significance indicated as ^****^ *p* < 0.0001.

To systematically benchmark therapeutic efficacy and clarify the contribution of MnO_2_ in vivo, DTBEI@CS was included as a MnO_2_‐free counterpart to DTBEI@CS@MnO_2_. Accordingly, DTBEI@CS@MnO_2_ was compared with MnO_2_ alone, DTBEI alone, DTBEI@CS, and the physical mixture DTBEI+CS+MnO_2_, with PBS‐treated mice serving as the control (referred to as the Model, MnO_2_, DTBEI, DTBEI@CS, DTBEI+CS+MnO_2_, and DTBEI@CS@MnO_2_ groups, respectively) (Figure 4D). Tumors in the Model and MnO_2_ groups exhibited comparable rapid and continuous growth, indicating limited anti‐tumor activity of MnO_2_ alone (Figure 4E,F and Figures S33–S36). Because of the immunogenicity of DTBEI, the DTBEI group produced a moderate inhibitory effect, slowing tumor development relative to the Model group without achieving complete suppression (Figure 4G and Figures S33–S36). A similar moderate effect was observed in the DTBEI@CS group (Figures S35 and S36), suggesting that CS coating alone was insufficient to provide the full therapeutic benefit in the absence of MnO_2_. The DTBEI+CS+MnO_2_ group also delayed tumor growth but remained less effective than the integrated DTBEI@CS@MnO_2_ formulation (Figure 4H and Figures S27–S30), indicating that simple physical mixing did not fully reproduce the therapeutic effect of the CS@MnO_2_ coating. In contrast, DTBEI@CS@MnO_2_ exhibited the most pronounced anti‐tumor effect, as reflected by sustained tumor growth suppression and regression of nearly all tumors to undetectable levels (Figure 4I and Figures S33–S36).

These therapeutic effects were further confirmed by histological analyses. Hematoxylin and eosin (H&E) staining revealed extensive disruption of tumor architecture in the DTBEI@CS@MnO_2_ group, while the PBS group showed comparatively preserved tissue morphology (Figure 4J). Terminal deoxynucleotidyl transferase‐mediated dUTP nick‐end labeling (TUNEL) assays showed a markedly increased proportion of apoptotic cells in tumors treated with DTBEI@CS@MnO_2_ (Figure 4K), while Ki67 staining demonstrated the lowest proliferative activity among all groups (Figure 4L). These results indicate that DTBEI@CS@MnO_2_ suppresses tumor growth more effectively than either its individual components or their physical mixture, while also promoting extensive apoptosis and inhibiting tumor cell proliferation. Finally, the therapeutic effect was validated in an orthotopic CT26 colon tumor model, where tumor fragments were surgically implanted into the cecal wall. Mice treated with DTBEI@CS@MnO_2_ showed no detectable tumor nodules at the implantation site, whereas prominent tumors were observed in the PBS group (Figure 4M). Overall, these results demonstrate that DTBEI@CS@MnO_2_ exerts potent anti‐tumor efficacy in both subcutaneous and orthotopic CT26 tumor models.

To determine whether tumor‐growth inhibition translated into a survival benefit, an independent cohort of CT26 tumor‐bearing mice was treated with PBS or DTBEI@CS@MnO_2_ using the same four‐dose regimen (*n* = 8). The median survival time was 25 days in the PBS group, whereas no survival events occurred in the DTBEI@CS@MnO_2_ group during the 30‐day observation period, and the median survival time was not reached. Kaplan‐Meier analysis showed that DTBEI@CS@MnO_2_ significantly prolonged survival compared with PBS (Figure S37), indicating that its pronounced tumor‐growth inhibition was accompanied by a significant survival benefit.

### 2.4 Personalized Postoperative Treatment With DTBEI@CS@MnO_2_

Because residual tumor foci often lead to postoperative recurrence in clinical practice, we further examined whether this strategy could be tailored for personalized postsurgical therapy. Mice bearing established CT26 tumors were subjected to incomplete surgical resection to model residual disease. The resected tumor tissues were promptly processed into a cell suspension and subsequently used to prepare an autologous DTBEI@CS@MnO_2_ formulation following the same protocol (Figure 4N).

This approach allows the therapeutic agent to closely match the antigenic profile of the residual tumor, thus providing a personalized immunotherapeutic option for preventing relapse.

Mice with residual tumors were then randomly allocated to receive intravenous injection of PBS or DTBEI@CS@MnO_2_. During the 12‐day observation period, mice treated with PBS showed obvious tumor regrowth, while DTBEI@CS@MnO_2_ significantly inhibited postsurgical recurrence (Figure 4O,P). Importantly, no obvious adverse effects were detected during the entire treatment period. Overall, these results demonstrate that personalized DTBEI@CS@MnO_2_ formulations can be quickly generated from tumor tissues and may serve as a promising strategy for postoperative immunotherapy against tumor relapse.

### 2.5 Biosafety Evaluation of DTBEI@CS@MnO_2_

The in vivo biosafety of DTBEI@CS@MnO_2_ was further evaluated in healthy mice, with a dose‐matched MnO_2_ group included as a material control. Healthy mice were intravenously injected with PBS, MnO_2_, or DTBEI@CS@MnO_2_, and the MnO_2_ group received an equivalent MnO_2_ dose to that contained in the DTBEI@CS@MnO_2_ formulation. To assess acute systemic inflammatory responses, serum levels of interferon‐beta (IFN‐β), IL‐6, and TNF‐α were measured at 6 h after injection. Compared with the PBS group, neither MnO_2_ nor DTBEI@CS@MnO_2_ induced significant increases in these cytokines (Figure S38), suggesting that MnO_2_‐containing materials did not trigger an obvious acute systemic inflammatory response under the tested conditions. To further examine whether MnO_2_‐containing materials induced off‐target cGAS‐STING pathway activation in normal tissues, liver, spleen, and lung tissues were collected at 6 h after intravenous injection for WB analysis. Compared with the PBS group, neither MnO_2_ nor DTBEI@CS@MnO_2_ caused obvious upregulation of phosphorylated stimulator of interferon genes (p‐STING), phosphorylated TANK‐binding Kinase 1 (p‐TBK1), or phosphorylated interferon regulatory factor 3 (p‐IRF3) in these tissues (Figures S39–S41), suggesting that the STING‐TBK1‐IRF3 signaling axis was not detectably activated in the examined normal organs. Serum biochemical analysis was then performed at the end of the 21‐day observation period. Biochemical indicators, including albumin (ALB), alanine aminotransferase (ALT), aspartate aminotransferase (AST), triglycerides (TG), uric acid (UA), creatine kinase (CK), and total bile acid (TBA), showed no significant differences among the PBS, MnO_2_, and DTBEI@CS@MnO_2_ groups (Figure S42), indicating no obvious systemic biochemical toxicity. Consistently, H&E staining of major organs, including the heart, liver, spleen, lung, and kidney, showed no apparent histopathological abnormalities, inflammatory infiltration, necrosis, or structural damage after treatment (Figure S43). In particular, no apparent pulmonary vascular occlusion or embolism‐like pathological changes were observed in lung sections. Collectively, these results indicate that intravenous administration of DTBEI@CS@MnO_2_ did not induce obvious acute systemic inflammation, detectable off‐target cGAS‐STING activation, or apparent normal tissue injury under the tested dose and 21‐day observation period.

### 2.6 DTBEI@CS@MnO_2_ Potentiated Anti‐tumor Immunity and Remodeled the Tumor Immune Microenvironment

To elucidate the in vivo immunomodulatory mechanisms of DTBEI@CS@MnO_2_, tumor tissues were collected 24 h after the third administration of different formulations to ensure sufficient material for downstream cellular and signaling analyses. Considering that the maturation of DCs represents a crucial link between innate immune sensing and the initiation of adaptive immune responses [30], we first investigated DC activation in tumors. Flow cytometric analysis revealed that DTBEI@CS@MnO_2_ effectively promoted DC maturation, as evidenced by the increased expression of CD80 and CD86 and the elevated proportion of CD80^+^CD86^+^ mature DCs relative to the PBS and DTBEI monotherapy groups (Figure 5A). These findings suggest enhanced intratumoral antigen presentation capacity, which may support subsequent T‐cell activation and priming.

**FIGURE 5:**
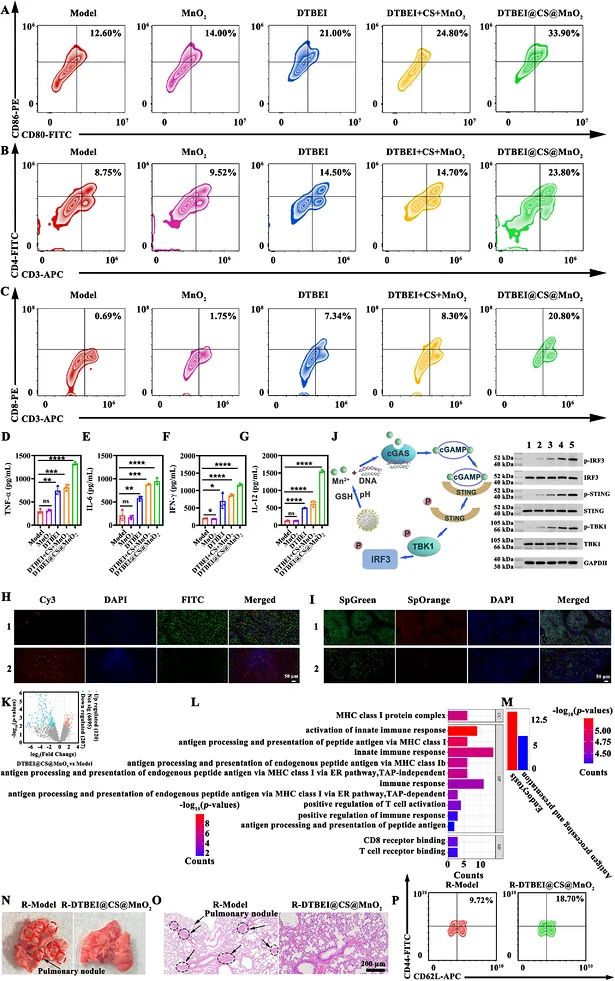
Mechanistic elucidation of the robust anti‐tumor efficacy of DTBEI@CS@MnO_2_. (A) Flow cytometric assessment of DC maturation via surface markers CD86 and CD80 in tumor tissues across different groups; The upper right quadrant defines the fraction of mature DCs (CD86^+^CD80^+^). (B) Flow cytometric quantification of CD3^+^CD4^+^ helper T cells in tumors (gated on CD3 and CD4), with the upper right quadrant representing the fraction of this subset following distinct treatments. (C) Flow cytometric profiling of intratumoral T lymphocyte subsets (gated on CD3 and CD8), where the upper right quadrant denotes the proportion of CD3^+^CD8^+^ CTLs. (D–G) Quantification of immunostimulatory cytokine secretion (i.e., TNF‐α, IL‐6, IFN‐γ, and IL‐12) in the mice after different treatments (*n* = 3). (H) Immunofluorescence staining of iNOS (a marker for M1‐type macrophages) and CD206 (a marker for M2‐type macrophages) in tumor tissues to assess tumor‐associated macrophage polarization following DTBEI@CS@MnO_2_ treatment. Scale bar = 100 µm. Red: iNOS (Cy3‐conjugated anti‐iNOS antibody); Green: CD206 (FITC‐conjugated anti‐CD206 antibody); Blue: Cell nuclei (DAPI counterstain). (I) Immunofluorescence staining for Foxp3^+^CD4^+^ regulatory T cells in tumor tissues of mice treated with DTBEI@CS@MnO_2_. Scale bar = 50 µm. Green: Foxp3 (SpGreen‐conjugated recombinant anti‐Foxp3 antibody); Red: CD4 (SpOrange‐conjugated recombinant anti‐CD4 antibody); Yellow: Merged signal of green and red channels. (J) Schematic illustration (left) and WB analysis (right) of cGAS‐STING pathway activation in tumor tissues (1: Model group; 2: MnO_2_ group; 3: DTBEI group; 4: DTBEI+CS+MnO_2_ group; 5: DTBEI@CS@MnO_2_ group). (K) Volcano plot depicting differential protein expression in tumor tissues between the DTBEI@CS@MnO_2_ and Model groups. (L) Enriched GO biological process terms for differentially expressed immune‐related proteins in tumor tissues. (M) Enriched KEGG pathways for differentially expressed immune‐related proteins in tumor tissues. (N) Representative digital images of lung tissues from mice in the R‐Model and R‐DTBEI@CS@MnO_2_ groups. Pulmonary metastatic nodules are demarcated by red dashed circles. (O) Representative H&E‐stained sections of lung tissues from mice after different treatments. Scale bar = 200 µm. Pulmonary nodules are delineated by black dashed circles. (P) Flow cytometric analysis of splenic CD44^+^CD62L^+^ double‐positive cells following homologous CT26 rechallenge. Data are presented as mean ± SD. Statistical analyses were performed using one‐way ANOVA; significance is indicated as ^*^ *p* < 0.05, ^**^ *p* < 0.01, ^***^ *p* < 0.001, or ^****^ *p* < 0.0001, and “ns” denotes no significance.

We then evaluated adaptive immune responses and found that DTBEI@CS@MnO_2_ increased the proportions of intratumoral CD3^+^CD4^+^ helper T cells and CD3^+^CD8^+^ cytotoxic T lymphocytes (CTLs) (Figure 5B,C). Functionally, CD8^+^ CTLs directly mediate antigen‐specific tumor cell killing, whereas CD4^+^ T cells support CTL expansion and persistence through immunoregulatory signaling [31, 32]. To further evaluate overall T‐cell effector activity, total IFN‐γ levels in tumor lysates were quantified using a commercial enzyme‐linked immunosorbent assay (ELISA) kit. The results showed DTBEI and DTBEI@CS moderately increased total IFN‐γ, whereas DTBEI@CS@MnO_2_ induced the highest IFN‐γ levels among all groups. The physical mixture DTBEI+CS+MnO_2_ also increased IFN‐γ, but to a lesser extent than DTBEI@CS@MnO_2_ (Figure S44). The highest IFN‐γ levels, together with the increased CD3^+^CD4^+^ and CD3^+^CD8^+^ T‐cell infiltration observed by flow cytometry, indicate enhanced T‐cell functional activation in the TME. Consistent with this, DTBEI@CS@MnO_2_ treatment markedly elevated the secretion of immunostimulatory cytokines, including TNF‐α, IL‐6, IFN‐γ, and IL‐12 (Figure 5D–G), further confirming its potent immune‐activating effect. Collectively, these findings suggest that DTBEI@CS@MnO_2_ enhances adaptive anti‐tumor immunity, which may contribute to its overall therapeutic efficacy.

In addition to activating effector immune cells, DTBEI@CS@MnO_2_ also alleviated immunosuppressive features within the TME. Immunofluorescence staining demonstrated an increase in iNOS^+^ M1‐like macrophages and a reduction in CD206^+^ M2‐like macrophages (Figure 5H), indicating a shift of tumor‐associated macrophage (TAM) polarization toward an anti‐tumor phenotype [33]. Meanwhile, DTBEI@CS@MnO_2_ treatment significantly reduced the proportion of Foxp3^+^CD4^+^ regulatory T cells (Tregs) (Figure 5I), which may help alleviate Treg‐mediated suppression of effector T‐cell function. This coordinated regulation of immunosuppressive cell populations, together with the activation of immune effector cells, helps reshape the tumor immune microenvironment and strengthens the anti‐tumor immune response.

Mechanistically, these phenotypic changes were associated with activation of the STING pathway, which serves as a key mediator of cytosolic DNA sensing and innate immune initiation. Upon activation, STING recruits TANK‐binding kinase 1 (TBK1), which subsequently promotes the phosphorylation of TBK1 and interferon regulatory factor 3 (IRF3). Phosphorylated IRF3 then translocates to the nucleus, where it induces type I interferon‐related programs and inflammatory mediator production, ultimately amplifying downstream immune responses [23]. WB showed that DTBEI@CS@MnO_2_ treatment increased the phosphorylation levels of STING, TBK1, and IRF3 in tumor tissues (Figure 5J), indicating activation of STING‐related signaling.

To further interpret these immune phenotypes at the molecular level, proteomic analysis identified 120 upregulated proteins and 207 downregulated proteins in the DTBEI@CS@MnO_2_ group relative to the Model group (Figure 5K), suggesting extensive remodeling of the tumor proteome. Gene Ontology (GO) analysis highlighted enrichment of biological processes related to antigen processing and presentation, innate immune activation, and MHC‐associated functions (Figure 5L). Consistently, Kyoto Encyclopedia of Genes and Genomes (KEGG) analysis revealed immune‐related pathways including antigen processing and presentation and endocytosis (Figure 5M). Together, these cellular, signaling, and omics results support that DTBEI@CS@MnO_2_ reshapes the immunosuppressive TME through coordinated regulation of innate sensing and antigen‐presentation pathways, ultimately facilitating anti‐tumor immune activation.

### 2.7 DTBEI@CS@MnO_2_‐Induced Immune Memory Suppressed Metastatic Rechallenge

Given that STING‐driven innate activation underpins durable adaptive immunity, we next evaluated whether DTBEI@CS@MnO_2_ could establish immune memory and protect against metastatic rechallenge. CT26 tumor‐bearing mice were treated according to the aforementioned regimen and, after completion of therapy, were intravenously rechallenged with homologous CT26 cells via tail vein injection (R‐DTBEI@CS@MnO_2_), with PBS‐treated mice serving as controls (R‐Model). Ten days after rechallenge, lungs were harvested for analysis. Macroscopic inspection and H&E staining revealed a markedly reduced pulmonary metastatic burden in the R‐DTBEI@CS@MnO_2_ group compared with the R‐Model group (Figure 5N,O), indicating effective protection against metastatic outgrowth. Consistently, flow cytometric analysis showed a significantly higher proportion of splenic CD44^+^CD62L^+^ double‐positive cells in the DTBEI@CS@MnO_2_‐treated group after homologous tumor rechallenge (Figure 5P and Figure S45). Together with the reduced pulmonary metastatic burden, these findings are consistent with an enhanced memory‐associated anti‐tumor immune response, which may contribute to limiting metastatic colonization following secondary tumor challenge.

### 2.8 Broad Applicability Across Tumor Models

The proposed strategy is modular and can be readily extended to different tumor types by using tumor‐matched cell templates. Using the same protocol as for DTBEI@CS@MnO_2_, we prepared 4T1‐derived (4T1‐DTBEI@CS@MnO_2_) and B16F10‐derived (B16F10‐DTBEI@CS@MnO_2_) constructs. Stepwise assembly was confirmed by zeta potential shifts after CS@MnO_2_ deposition (Figures S46 and S47), while SEM images revealed a distinct coating on both constructs (Figures S48 and S49). In homologous tumor models, 4T1‐DTBEI@CS@MnO_2_ markedly suppressed tumor growth and induced pronounced tumor regression (Figure 6A–C and Figure S50). Notably, in the poorly immunogenic B16F10 model, B16F10‐DTBEI@CS@MnO_2_ also significantly inhibited tumor progression (Figures S51–S54). Mechanistically, both constructs elicited consistent immune activation, characterized by enhanced intratumoral DC maturation (Figure 6D and Figure S55) and increased infiltration of CD3^+^CD4^+^ and CD3^+^CD8^+^ T cells (Figure 6E,F and Figures S56 and S57). Besides, total IFN‐γ levels in 4T1 tumor lysates were also quantified using a commercial ELISA kit. Consistent with increased CD3^+^CD4^+^ and CD3^+^CD8^+^ T‐cell infiltration, the 4T1‐DTBEI@CS@MnO_2_ group exhibited markedly elevated IFN‐γ levels compared with the Model group, supporting enhanced T‐cell effector function in the TME (Figure S58). Furthermore, DTBEI@CS@MnO_2_ treatment markedly elevated the secretion of other immunostimulatory cytokines, including TNF‐α, IL‐6, IFN‐γ, and IL‐12 (Figure 6G–J and Figure S59). In parallel, the tumor immune microenvironment was remodeled toward an anti‐tumor state, as evidenced by increased M1‐like and decreased M2‐like macrophage markers (Figure 6K and Figure S60) together with reduced Foxp3^+^CD4^+^ Tregs (Figure 6L and Figure S61). Collectively, these results indicate that the tumor cell‐templated platform can be extended to different tumor models.

**FIGURE 6:**
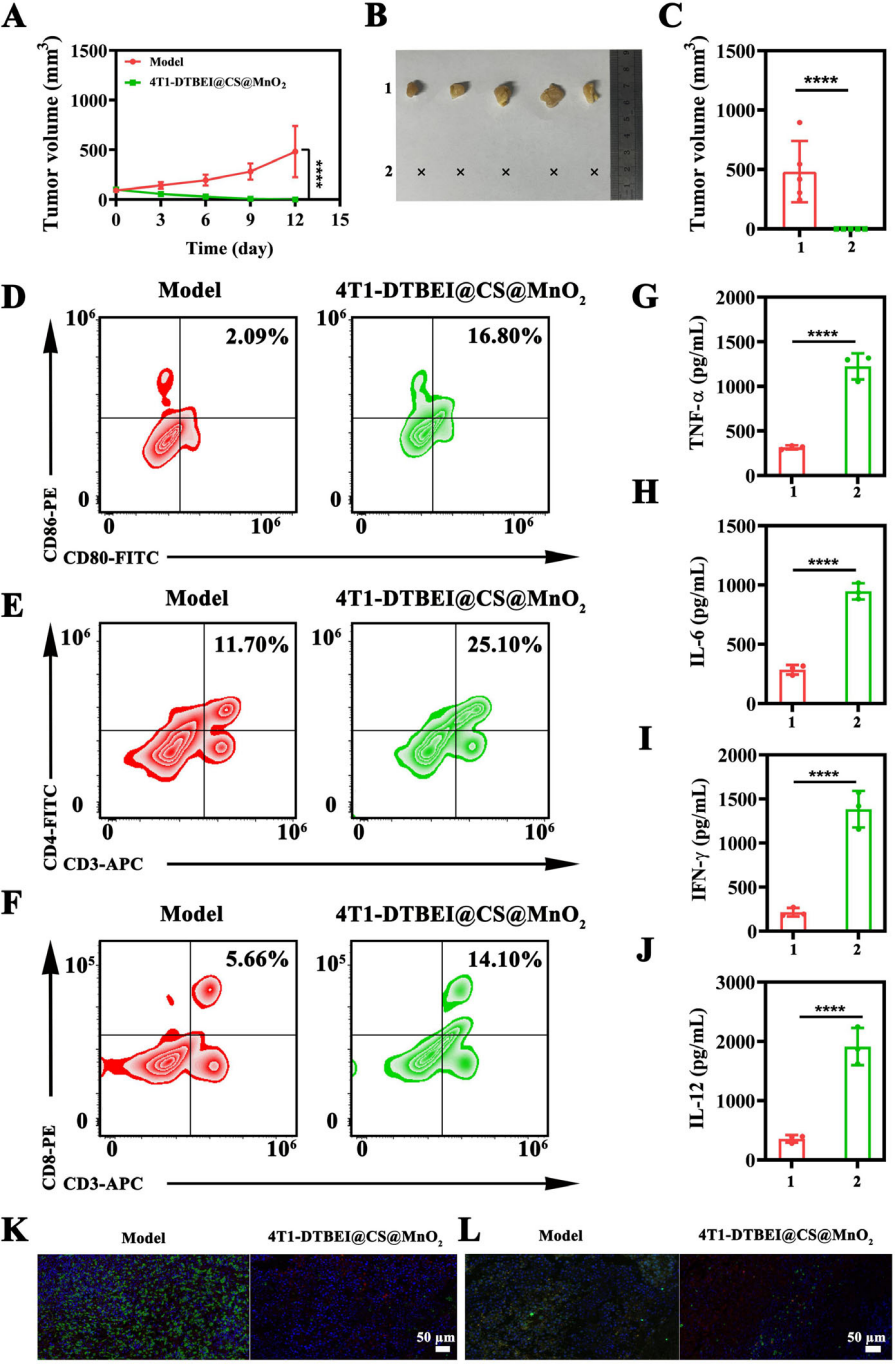
Broad applicability of tumor cell‐templated immune activators in the 4T1 breast tumor model. (A) Time‐dependent 4T1 tumor volume changes in mice post 4T1‐DTBEI@CS@MnO_2_ treatment (*n* = 5). (B) Digital photograph of excised 4T1 tumors from 4T1‐bearing mice at the end of treatment. 1: Model; 2: 4T1‐DTBEI@CS@MnO_2_ (*n* = 5). (C) Ex vivo 4T1 tumor size quantification at endpoint (*n* = 5). (D) Flow‐cytometric quantification of DC maturation (CD80^+^CD86^+^) in 4T1 tumors; The upper right quadrant indicates the percentage of mature DC. (E) Flow‐cytometric quantification of helper T cells (CD3^+^CD4^+^) in 4T1 tumors of 4T1‐DTBEI@CS@MnO_2_‐treated mice. The upper right quadrant shows the percentage of CD3^+^CD4^+^ T cells. (F) Flow‐cytometric quantification of CTLs (CD3^+^CD8^+^) in 4T1 tumors of 4T1‐DTBEI@CS@MnO_2_‐treated mice. The upper right quadrant indicates the percentage of CD3^+^CD4^+^ T cells from mice treated with 4T1‐DTBEI@CS@MnO_2_. (G–J) Cytokine (i.e., TNF‐α, IL‐6, IFN‐γ, IL‐12) concentrations in 4T1 tumor‐bearing mice after treatment with 4T1‐DTBEI@CS@MnO_2_ (*n* = 3). 1: Model; 2: 4T1‐DTBEI@CS@MnO_2_. (K) Representative immunofluorescence images of iNOS (M1 macrophage marker) and CD206 (M2 macrophage marker) in 4T1 tumors after 4T1‐DTBEI@CS@MnO_2_ treatment. Scale bar, 50 µm. Red: iNOS (Cy3‐anti‐iNOS); Green: CD206 (FITC‐anti‐CD206); Blue: nuclei (DAPI). (L) Immunofluorescence staining of Foxp3^+^CD4^+^ regulatory T cells in tumors from 4T1‐DTBEI@CS@MnO_2_‐treated mice. Scale bar, 50 µm. Green: Foxp3 (SpGreen‐recombinant‐anti‐Foxp3); Red: CD4 (SpOrange‐recombinant anti‐CD4); Yellow: merged signal. Data are mean ± SD. Statistical analysis by Student's *t*‐test; significance: ^****^ *p* < 0.0001.

## 3 Conclusions

In this work, we developed a modular electrostatic co‐engineering strategy to convert ICD‐primed DTBs into a TME‐responsive whole‐cell immunotherapeutic platform. Tumor cells were first preloaded with ICG and sequentially coated with a CS interlayer and MnO_2_ adsorbates to form a CS@MnO_2_ predominantly surface‐associated CS@MnO_2_ coating. Upon 808 nm laser irradiation, ICG‐mediated photothermal/photodynamic effect induced ICD, generating DTBEI while preserving the outer coating. In the acidic and GSH‐enriched TME, CS@MnO_2_ disassembly generated Mn^2^^+^ to potentiate DNA‐triggered cGAS‐STING signaling. DTBEI simultaneously supplied tumor antigens and ICD‐associated DNA/DAMPs, enabling amplified innate sensing to drive stronger antigen cross‐presentation and T‐cell priming. Consequently, DTBEI@CS@MnO_2_ elicited robust anti‐tumor immunity in both subcutaneous and orthotopic CT26 models and effectively suppressed postoperative recurrence. Rechallenge experiments further suggested the establishment of memory‐associated anti‐tumor immunity, accompanied by increased CD62L^+^CD44^+^ memory‐phenotype T cells and reduced pulmonary metastatic burden. Importantly, this modular strategy could be extended to additional tumor cell templates, enabling effective tumor control and immune activation in 4T1 and B16F10 models. Moreover, DTBEI@CS@MnO_2_ exhibited good in vivo biocompatibility and favorable stability under physiological and storage conditions. Based on this design, no additional laser irradiation was applied during in vivo treatment, allowing us to evaluate the intrinsic TME‐responsive immunotherapeutic effect of DTBEI@CS@MnO_2_. Laser‐assisted treatment may further enhance local tumor suppression in accessible tumors and will be explored in future studies. Collectively, this work establishes a materials‐enabled paradigm that integrates antigen‐rich whole‐cell substrates with innate immune potentiation, providing a promising and personalizable approach for cancer immunotherapy.

## 4 Experimental Section

### 4.1 Materials

CS, MnCl_2_, NaOH, H_2_O_2_, and GSH were purchased from Aladdin Industries (Shanghai, China). Cell Counting Kit‐8 (CCK‐8) for assessing cell proliferation and cytotoxicity was procured from Solarbio Life Sciences (Beijing, China). RIPA lysis buffer (cat. no. G2002‐100ML), Protease inhibitor cocktail (50 ×, cat. no. G2006‐250UL), Phenylmethylsulfonyl fluoride (100 mm) (cat. no. G2008‐1ML), prestained protein marker VII (8–195 kDa) (cat. no. G2087‐250UL), Tris‐buffered saline with Tween (cat. no. G2150‐1L), primary antibody dilution buffer (cat. no. G2025‐100ML), and secondary antibody dilution buffer for WB (cat. no. G2009‐100ML) were all purchased from Servicebio (Wuhan, China). BCA protein quantification kit (cat. no. BL521A) was procured from Biosharp (Hefei, China). MOPS‐SDS running buffer (cat. no. F00004Gel) and FuturePAGE precast protein gel (cat. no. ET12420Gel) were all purchased from ACE (Shanghai, China). Ponceau S staining solution (cat. no. P0022), Coomassie brilliant blue rapid staining solution (cat. no. P0017), QuickBlock western blocking buffer (cat. no. P0252), QuickBlock western primary antibody dilution buffer (cat. no. P0256), QuickBlock western secondary antibody dilution buffer (cat. no. P0258), and western rapid transfer buffer (powder, cat. no. P0575) were all purchased from Beyotime Biotechnology (Shanghai, China). PVDF membrane (cat. no. IPVH00010) was procured from Merck Millipore (Darmstadt, Germany). Meilunbio Keke ultra—sensitive ECL luminescence solution (cat. no. MA0186) was procured from Meilunbio (Dalian, China). ELISA kits for measuring TNF‐α, IFN‐γ, IL‐6, and IL‐12 levels were all acquired from Bost Biotechnology (Wuhan, China). APC‐anti‐mouse CD3 (Cat. #100236, 100 µg), FITC‐anti‐mouse CD80 (Cat. #14706, 500 µg), PE‐anti‐mouse CD86 (Cat. #159204, 50 µg), FITC‐anti‐mouse CD4 (Cat. #100406, 50 µg), and PE‐anti‐mouse CD8 (Cat. #162304, 50 µg) were all purchased from Biolegend (San Diego, USA).

### 4.2 Cell Lines and Animals

All cell lines used in this study were purchased from Procell Life Science & Technology Co., Ltd. (Wuhan, China). CT26 and B16F10 cells were grown in RPMI 1640 medium, whereas 4T1 cells were cultured in DMEM. Both media were supplemented with 10% FBS and 1% penicillin‐streptomycin solution. Cell cultures were incubated at 37°C in a humidified incubator containing 5% CO_2_.

Male C57BL/6 mice and female BALB/c mice, both aged five weeks, were acquired from Gempharmatech (Jiangsu, China). The mice were maintained in the Animal Science Laboratory Center of Nanchang University under controlled environmental conditions: a 12‐h light/dark cycle, relative humidity ranging from 40% to 60%, and a stable temperature of 22°C ± 1°C. All animal experiments were carried out strictly in accordance with protocols authorized by the Animal Care and Use Committee of Nanchang University (approval number: NCULAE‐20260112001).

### 4.3 LBTB Preparation

1 × 10^7^ CT26 cells were collected by centrifugation, and the supernatant culture medium was carefully aspirated. The cell pellet was rinsed twice with ice‐cold PBS to remove residual medium, then resuspended in 1 mL PBS in a sterile centrifuge tube. Subsequently, 150 µL RIPA lysis buffer was added to the cell suspension to obtain DTB.

### 4.4 DTBEI Preparation

1 × 10^7^ CT26 cells were seeded in 6‐well plates and incubated with ICG solution (final concentration: 8 µg/mL) at 37°C for 2 h. After incubation, the ICG‐containing medium was discarded, and the cells were washed three times with ice‐cold PBS to remove non‐uptaken ICG. The washed cells were then exposed to an 808 nm laser (power density: 1.0 W/cm^2^) for 8 min to induce the generation of DTBEI.

### 4.5 Detection of ICD‐Associated DAMPs After Laser Irradiation

To directly evaluate ICD‐associated DAMPs during DTBEI preparation, CT26 cells were incubated with ICG under the same conditions for DTBEI fabrication and then irradiated with an 808 nm laser. Untreated CT26 cells, CT26 cells treated with 808 nm laser alone, CT26‐ICG cells without laser irradiation, and DOX‐treated CT26 cells were used as untreated, laser‐only, ICG‐only, and positive ICD controls, respectively. All groups were processed under equivalent cell numbers. CRT‐associated extracellular levels were measured at 4 h after irradiation or DOX treatment using a calreticulin assay kit according to the manufacturer's instructions. Extracellular ATP secretion was measured at 2 h after irradiation or DOX treatment using a luciferase‐based ATP assay kit. HMGB1 release in the culture supernatants was quantified at 24 h after irradiation or DOX treatment using an HMGB1 ELISA kit. These assays were performed to directly assess ICD‐associated DAMP generation after 808 nm laser‐triggered ICG treatment.

### 4.6 Assessment of Immune Responses Elicited by LBTB or DTBEI

Splenocytes were isolated from normal mice using a standardized protocol as follows: fresh spleens were minced and ground gently, then passed through a 200‐mesh cell strainer to obtain single‐cell suspensions. After washing twice with sterile PBS, red blood cells in the suspension were lysed using 1 mL lysis buffer. The remaining cells were resuspended in RPMI 1640 medium and incubated for 24 h at 37°C in a 5% CO_2_ incubator. The splenocytes were plated into 6‐well culture plates at 3 × 10^6^ cells per well. Cells were then treated with LBTB or DTBEI and incubated for an additional 24 h. For DC phenotyping, cells were harvested, washed with PBS containing 2% FBS, and stained with FITC‐anti‐mouse CD80 and PE‐anti‐mouse CD86 for 30 min at 4°C in the dark. Concurrently, the leftover cell suspensions were stained with APC‐anti‐mouse CD3, FITC‐anti‐mouse CD4, and PE‐anti‐mouse CD8 for T cell subset analysis. The concentrations of IL‐6, IL‐12, IFN‐γ, and TNF‐α in the cell culture supernatants were measured using commercially available ELISA kits according to the manufacturer's instructions.

### 4.7 Preparation and Characterization of DTBEI@CS@MnO_2_

CT26 cells were first suspended in 1 mL of RPMI 1640 medium. ICG was added to a final concentration of 8 µg/mL, and the cells were incubated for 2 h. Next, CS was introduced at varying concentrations (i.e., 0, 50, 100, 150, 200, and 250 µg/mL). The mixtures were further incubated at 37°C for different durations (i.e., 0, 5, 10, 15, and 20 min) to form distinct CT26‐ICG@CS. Based on the changes in zeta potential, the optimal conditions for preparing CT26‐ICG@CS were determined to be a CS concentration of 200 µg/mL and an incubation time of 15 min.

Subsequently, MnO_2_ was introduced into the system at 0.435 mg/mL, followed by incubation at 37°C for 0, 10, 20, 40, or 80 min to generate different CT26‐ICG@CS@MnO_2_ formulations. Zeta potential analysis was performed again and showed that a 40 min incubation time was optimal for MnO_2_ incorporation during the preparation of CT26‐ICG@CS@MnO_2_. The resulting CT26‐ICG@CS@MnO_2_ was then irradiated with an 808 nm laser (1 W/cm^2^) for 8 min to yield the final DTBEI@CS@MnO_2_. Morphology was characterized using scanning electron microscopy (SEM, FEI Quanta Feg 250, USA) and transmission electron microscopy (TEM, JEM‐F200, JEOL, Japan). Zeta potential was measured with a Zetasizer Pro analyzer (Malvern Panalytical, UK). Elemental composition was determined by energy‐dispersive x‐ray spectroscopy (EDS, FEI Quanta Feg 250, USA) and x‐ray photoelectron spectrometer (XPS, ESCALAB 250Xi, Thermo Fisher Scientific, USA).

### 4.8 Preparation of DTBEI@CS

DTBEI@CS was prepared using the same procedure as DTBEI@CS@MnO_2_, except that the MnO_2_ adsorption step was omitted. Briefly, CT26 cells were first incubated with ICG, followed by CS coating under the optimized conditions described above. The resulting CT26‐ICG@CS was then irradiated with an 808 nm laser at 1.0 W/cm^2^ for 8 min to induce ICD and generate DTBEI@CS. For all comparative experiments, the DTBEI dose was normalized by tumor‐cell equivalents.

### 4.9 CLSM Analysis of CS Localization on CT26‐ICG Cells

CT26‐ICG cells were incubated with Cy3‐labeled chitosan (Cy3‐CS) at a final concentration of 200 µg/mL for 15 min at 37°C under the same optimized conditions used for CT26‐ICG@CS preparation. After incubation, the cells were washed three times with PBS to remove unbound Cy3‐CS. The plasma membrane was subsequently stained with the lipophilic membrane dye DiO according to the manufacturer's instructions, and cell nuclei were counterstained with DAPI. Fluorescence images and Z‐stack series were acquired using a CLSM. Cy3‐CS, DiO‐labeled plasma membrane, and DAPI‐stained nuclei were visualized in the red, green, and blue channels, respectively. The spatial distribution of Cy3‐CS relative to the plasma membrane and nucleus was evaluated using representative X‐Y optical sections and Z‐stack images.

### 4.10 Quantification of CS and MnO_2_ Loading

For CS quantification, CS was fluorescently labeled with fluorescein isothiocyanate (FITC) through the reaction between the isothiocyanate group of FITC and the amino groups of CS under mild conditions. Free FITC was thoroughly removed by dialysis. A fluorescence calibration curve was then established using a series of FITC‐labeled CS standards with known concentrations, including 0, 50, 100, 200, and 300 µg/mL. The integrated fluorescence area showed good linearity with CS concentration, with the calibration equation of y = 115.63x – 450.57 and R^2^ = 0.9954. Meanwhile, DTBEI@CS‐FITC@MnO_2_ was prepared using FITC‐labeled CS under the same assembly conditions as those used for DTBEI@CS@MnO_2_. The supernatant and material‐associated fractions were separately collected, and their FITC‐labeled CS amounts were calculated from the calibration curve. The CS loading efficiency was calculated as [C_material‐associated_/(C_supernatant_ + C_material‐associated_)] × 100%.

For MnO_2_ quantification, the purified DTBEI@CS@MnO_2_ formulation was completely digested with acid, and the Mn concentration was determined by ICP‐MS. The MnO_2_ amount was calculated from the measured Mn content using the molecular‐weight conversion factor of MnO_2_/Mn: C_MnO2_ = C_Mn​_ ​ × (M_Mn_​/M_MnO2_​​​).

### 4.11 HMGB1 Leakage Assay

To evaluate the DAMP‐retention capability of the CS@MnO_2_ coating, free DTBEI, DTBEI@CS, DTBEI@CS@MnO_2_, and CT26‐ICG@CS@MnO_2_ without laser irradiation were incubated under physiological pH 7.4 conditions. At predetermined time points of 0, 4, 8, 12, and 24 h, the supernatants were collected, and HMGB1 levels were quantified using an HMGB1 ELISA kit according to the manufacturer's instructions. The HMGB1 leakage ratio was calculated as the amount of HMGB1 in the supernatant divided by the total HMGB1 content. To further assess TME‐responsive DAMP release, DTBEI@CS@MnO_2_ was also incubated under acidic pH 6.5 conditions in the presence of 1 mm GSH, and HMGB1 release was quantified at the same time points.

### 4.12 Flow Cytometric Analysis of Antibody‐Accessible CRT

To evaluate the effect of CS‐based encapsulation on CRT accessibility, untreated CT26 cells, CT26‐ICG cells without laser irradiation, free DTBEI, DTBEI@CS, and DTBEI@CS@MnO_2_ were prepared using equivalent cell numbers. DTBEI@CS@MnO_2_ was incubated either under pH 7.4 conditions or under pH 6.5 conditions in the presence of 1 mm GSH for 24 h at 37°C. After washing with PBS, the samples were directly stained with FITC‐conjugated anti‐CRT antibody for 1 h at 4°C in the dark. Flow cytometry data were analyzed using a sequential gating strategy. Cell‐body‐sized events were selected based on FSC‐A/SSC‐A, followed by exclusion of aggregates using FSC‐H/FSC‐A. CRT‐positive events were quantified within the gated singlet population. The CRT‐positive threshold was defined using matched unstained controls and was applied consistently to all groups.

### 4.13 Assessment of Storage‐Associated Protein Changes in DTBEI and DTBEI@CS@MnO_2_

DTBEI and DTBEI@CS@MnO_2_ were independently prepared in three biological batches, each derived from a separate cell culture and formulation preparation. Freshly prepared samples were used as day‐0 baseline controls, and the corresponding samples were stored at 37°C for the indicated periods.

For label‐free quantitative proteomic analysis, fresh and day‐7 DTBEI and DTBEI@CS@MnO_2_ samples were independently processed for protein extraction, tryptic digestion, liquid chromatography‐tandem mass spectrometry (LC‐MS/MS), and data analysis. For rank‐abundance analysis, the label‐free quantification abundance of each membrane‐associated protein was normalized to the mean abundance of the corresponding protein in fresh DTBEI at day 0. Selected representative membrane and cell‐surface proteins were visualized by heatmap analysis.

For time‐course SDS‐PAGE analysis, samples from each biological batch were divided into equal aliquots containing the same number of tumor‐cell equivalents and analyzed at days 0, 1, 3, and 7. At each time point, equal tumor‐cell‐equivalent amounts were lysed, separated by SDS‐PAGE, and stained with Coomassie brilliant blue. The background‐subtracted integrated intensity of each entire lane was quantified using ImageJ and normalized to that of the corresponding fresh day‐0 sample.

For WB analysis, fresh and day‐7 DTBEI and DTBEI@CS@MnO_2_ samples were analyzed using equal tumor‐cell‐equivalent loading. The membranes were probed with antibodies against HMGB1, HSP90, EpCAM, integrin β1, and glyceraldehyde‐3‐phosphate dehydrogenase (GAPDH). Protein bands were visualized by enhanced chemiluminescence under non‐saturating exposure conditions and quantified using ImageJ after background subtraction. Target‐protein signals were normalized to the corresponding GAPDH signals.

### 4.14 Dual pH/GSH‐Triggered DTBEI Release Assay

To systematically evaluate the effects of acidic pH and GSH on the disassembly of DTBEI@CS@MnO_2_, DTBEI release was examined using a complete 2 × 2 orthogonal design. Four release media were prepared: pH 7.4 without GSH, pH 6.5 without GSH, pH 7.4 with 1 mm GSH, and pH 6.5 with 1 mm GSH. DTBEI@CS@MnO_2_ samples containing an equivalent amount of DTBEI were dispersed in each release medium and incubated at 37°C under gentle shaking. At predetermined time points, the samples were gently centrifuged, and the supernatants containing released DTBEI‐associated proteins were collected. The amount of released DTBEI was quantified using a BCA protein assay kit according to the manufacturer's instructions. The protein content in each collected supernatant was measured, and the cumulative DTBEI release percentage was calculated as follows: Cumulative DTBEI release (%) = (protein amount in the supernatant at each time point/total protein amount of DTBEI loaded in DTBEI@CS@MnO_2_) × 100%.

### 4.15 MB Bleaching Assay

The redox‐responsive activity of DTBEI@CS@MnO_2_ was evaluated using a MB bleaching assay. Briefly, DTBEI@CS@MnO_2_ with an equivalent Mn concentration of 0.5 mm was dispersed in 25 mm NaHCO_3_/5% CO_2_ buffer (pH 7.4), followed by incubation with or without 1 mm GSH for 15 min under gentle shaking. After centrifugation, the supernatant was collected for subsequent analysis. The following groups were prepared: MB, MB+H_2_O_2_, MB+GSH, MB+H_2_O_2_+DTBEI@CS@MnO_2_, MB+GSH+DTBEI@CS@MnO_2_, and MB+H_2_O_2_+GSH+DTBEI@CS@MnO_2_. MB and H_2_O_2_ were added at final concentrations of 10 µg/mL and 8 mm, respectively, where applicable. After incubation at 37°C for 30 min, the absorbance at 665 nm was recorded. The decrease in MB absorbance was used to evaluate the oxidative activity of DTBEI@CS@MnO_2_ under different redox conditions.

### 4.16 In vitro BMDC Maturation

Bone marrow cells were isolated from mouse femurs and tibias and cultured in complete RPMI 1640 medium supplemented with granulocyte‐macrophage colony‐stimulating factor and interleukin‐4 to generate BMDCs. On day 6 or 7, non‐adherent and loosely adherent cells were collected and seeded into 6‐well plates. The BMDCs were then treated with samples collected from MnO_2_, DTBEI, DTBEI@CS, DTBEI+CS+MnO_2_, or DTBEI@CS@MnO_2_ after incubation under pH 6.5 or pH 7.4 conditions. After 24 h, cells were harvested and stained with anti‐CD80 and anti‐CD86 antibodies. BMDC maturation was assessed by flow cytometry. For analysis, debris was first excluded based on FSC/SSC characteristics, followed by singlet selection using FSC‐A vs. FSC‐H. CD80 and CD86 expression was then analyzed within the selected BMDC culture‐derived cell population, and CD80^+^CD86^+^ cells were quantified as mature BMDCs.

### 4.17 In vitro T‐cell Population and Cytokine Analysis

For downstream immune‐response analysis, murine splenocytes were seeded into 6‐well plates (3 × 10^6^ cells/well) and treated with samples collected from MnO_2_, DTBEI, DTBEI@CS, DTBEI+CS+MnO_2_, or DTBEI@CS@MnO_2_ after incubation under pH 6.5 or pH 7.4 conditions. After 24 h, cells were harvested for flow cytometric analysis. T‐cell populations were analyzed by staining with APC‐anti‐mouse CD3, FITC‐anti‐mouse CD4, and PE‐anti‐mouse CD8 antibodies. For T‐cell analysis, lymphocytes were first selected according to FSC/SSC characteristics, followed by singlet selection. CD3^+^ T cells were then gated and further analyzed for CD4^+^ and CD8^+^ populations to quantify CD3^+^CD4^+^ and CD3^+^CD8^+^ T cells, respectively. The culture supernatants were collected, and the levels of IFN‐γ, IL‐6, TNF‐α, and IL‐12 were measured using ELISA kits according to the manufacturers’ instructions. In parallel, the treated splenocytes were collected and lysed, and total IFN‐γ levels in the splenocyte lysates were quantified using an ELISA kit.

### 4.18 In vivo Distribution of DTBEI@CS@MnO_2_ in CT26 Tumor‐Bearing Mice

CT26 tumors were established by subcutaneous inoculation of CT26 cells (5 × 10^6^ cells/ mouse) into the right flank of BALB/c mice. To minimize autofluorescence, mice were fed a fluorophore‐free diet for 1 week prior to imaging. When tumor volumes reached ∼100 mm^3^, mice were intravenously injected with DTBEI@CS@MnO_2_ (200 µL) via the tail vein. In vivo fluorescence distribution was monitored using a second near‐infrared (NIR‐II) imaging system (DeepVision, Nirmidas Biotech Inc., USA) at 0, 6, 8, 10, 48, and 72 h post‐injection.

### 4.19 In vitro Stability and In vivo Biodistribution Study Using DiD‐Labeled DTBEI@CS@MnO_2_

To further evaluate the membrane/cell‐body‐associated biodistribution of DTBEI@CS@MnO_2_, DiD, a lipophilic membrane‐associated fluorescent dye, was used for additional labeling. Briefly, CT26 cells were first incubated with ICG under the same conditions used for DTBEI preparation. After washing with PBS, the cells were incubated with DiD working solution (5 µm) at 37°C for 20 min in the dark. The labeled cells were washed three times with PBS to remove excess free dye, followed by CS/MnO_2_ assembly and 808 nm laser irradiation to obtain DiD‐labeled DTBEI@CS@MnO_2_.

Before in vivo imaging, the labeling stability of DiD‐labeled DTBEI@CS@MnO_2_ was evaluated under serum‐containing conditions. DiD‐labeled DTBEI@CS@MnO_2_ was incubated in RPMI 1640 medium containing 10% FBS at 37°C. At predetermined time points of 0, 12, 24, 48, and 72 h, the samples were centrifuged, and the fluorescence intensities of the supernatant and pellet fractions were measured separately. The DiD leakage ratio was calculated as follows: DiD leakage ratio (%) = F_supernatant_/(F_supernatant_ + F_pellet_) × 100%.

For in vivo fluorescence imaging, CT26 tumor‐bearing mice were randomly divided into three groups (*n* = 3) and intravenously injected with PBS, free DiD, or DiD‐labeled DTBEI@CS@MnO_2_ through the tail vein. The DiD‐labeled DTBEI@CS@MnO_2_ group received 1 × 10^6^ DTBEI cell equivalents in 100 µL PBS per mouse, and the free DiD group received an equivalent DiD amount with comparable fluorescence intensity. In vivo fluorescence imaging was performed using the NIR‐II imaging system at 0, 6, 10, 48, and 72 h post‐injection. To further evaluate tissue distribution, mice were sacrificed at 10 h post injection. Tumors, lymph nodes, and major organs, including the heart, liver, spleen, lung, and kidney, were harvested for ex vivo fluorescence imaging.

### 4.20 Optimizing the DTBEI Payload in DTBEI@CS@MnO_2_ to Maximize CT26 Tumor Suppression​

The identical CT26 cell line used to prepare DTBEI@CS@MnO_2_ was also used to establish subcutaneous tumors. CT26 cells injected into the right thigh produced tumors that were allowed to reach approximately 100 mm^3^, after which mice were randomized into four groups (*n* = 5) and given a single injection of either PBS or DTBEI@CS@MnO_2_ prepared from the equivalent of 1 × 10^5^, 1 × 10^6^, or 1 × 10^7^ CT26 cells (designated as PBS group, 1 × 10^5^ group, 1 × 10^6^ group, and 1 × 10^7^ group, respectively). Tumor volumes were monitored every third day for 12 days using the formula (width^2^ × length)/2.

### 4.21 Optimization of the Administration Frequency of DTBEI@CS@MnO_2_ to Maximize CT26 Tumor Suppression

A homologous CT26 subcutaneous tumor model was established by subcutaneous inoculation of CT26 cells into the right flank of female BALB/c mice. When tumor volumes reached ∼100 mm^3^, mice were randomized into four groups (*n* = 5) and intravenously injected (tail vein) with PBS or DTBEI@CS@MnO_2_ (DTBEI dose normalized to 1 × 10^6^ CT26 tumor‐cell equivalents per injection). DTBEI@CS@MnO_2_ was administered every 3 days for a total of 2, 3, or 4 injections. Tumor volume was recorded at 3‐day intervals.

### 4.22 Anti‐Tumor Efficacy of DTBEI@CS@MnO_2_ in Subcutaneous CT26 Tumor‐Bearing Mice

CT26 subcutaneous tumor model was established by subcutaneous inoculation of CT26 cells (5 × 10^6^ cells/mouse) into the right flank of BALB/c mice. When tumor volumes reached ∼100 mm^3^, mice were randomized into six groups and intravenously injected with PBS, MnO_2_, DTBEI, DTBEI@CS, DTBEI+CS+MnO_2_, or DTBEI@CS@MnO_2_ (DTBEI dose normalized to 1 × 10^6^ tumor‐cell equivalents per injection). Treatments were administered every 3 days for a total of 4 injections. Tumor volumes were measured every 3 days and calculated as (length × width^2^)/2.

For mechanistic immune analysis, a separate set of CT26 tumor‐bearing mice received the same treatment schedule with PBS, MnO_2_, DTBEI, DTBEI+CS+MnO_2_, or DTBEI@CS@MnO_2_. At 24 h post‐third injection, five mice from each group were euthanized to harvest tumors and peripheral blood serum. Tumor cell suspensions were prepared by homogenizing the collected tumor tissues and filtering through cell sieves. For DC detection, these suspensions were stained with anti‐mouse CD11c, FITC‐anti‐mouse CD80, and PE‐anti‐mouse CD86. For T cell profiling, the remaining cell suspensions were stained with APC‐anti‐mouse CD3, FITC‐anti‐mouse CD4, and PE‐anti‐mouse CD8. Levels of relevant cytokines (i.e., TNF‐α, IL‐6, IFN‐γ, and IL‐12) were quantified using corresponding ELISA kits.

### 4.23 DTBEI@CS@MnO_2_‐Based Personalized Postoperative Therapy After Incomplete Resection

Tumor models were generated by subcutaneous inoculation of CT26 cells into the right thigh of mice. Following incomplete surgical resection, residual tumor tissue was homogenized and filtered to obtain cell suspensions from which DTBEI@CS@MnO_2_ was synthesized according to the protocol outlined in the “*Preparation and characterization of DTBEI@CS@MnO_2_*” section. Concurrently, the mice were randomly assigned to two groups that received intravenous injections of either PBS or DTBEI@CS@MnO_2_ every three days for a total of four injections. Tumor volumes were monitored at 3‐day intervals.

### 4.24 In vivo Biosafety Evaluation of DTBEI@CS@MnO_2_

Healthy BALB/c mice were randomly divided into three groups (*n* = 3) and intravenously injected with PBS, MnO_2_, or DTBEI@CS@MnO_2_. The MnO_2_ group received an equivalent MnO_2_ dose to that contained in the DTBEI@CS@MnO_2_ formulation. At 6 h after injection, blood samples were collected to measure serum IFN‐β, IL‐6, and TNF‐α levels. In parallel, liver, spleen, and lung tissues were harvested at 6 h after injection for WB analysis of p‐STING, p‐TBK1, and p‐IRF3. At the end of the 21‐day observation period, blood samples were collected for serum biochemical analysis, including ALB, ALT, AST, TG, UA, CK, and TBA. Major organs, including the heart, liver, spleen, lung, and kidney, were harvested for H&E staining.

### 4.25 In vivo Protection Against Metastatic Rechallenge Induced by DTBEI@CS@MnO_2_

A subcutaneous CT26 tumor model was established by inoculating CT26 cells (5 × 10^6^ cells/mouse) into the right flank of BALB/c mice. When tumors reached ∼100 mm^3^, mice were randomized into two groups (*n* = 5) and intravenously injected (tail vein) with PBS or DTBEI@CS@MnO_2_ (DTBEI dose normalized to 1 × 10^6^ tumor‐cell equivalents per injection) every 3 days for a total of 4 injections. Three days after the last treatment, mice were intravenously rechallenged with homologous CT26 cells (5 × 10^6^ cells/mouse) to establish an experimental lung colonization model. Ten days after rechallenge, lungs were collected for gross assessment of metastatic nodules and H&E staining. And spleens were harvested and processed into single‐cell suspensions for flow cytometric analysis of CD44 and CD62L expression.

### 4.26 DTBEI@CS@MnO_2_‐Based Personalized Therapy in 4T1 and B16F10 Subcutaneous Tumor Models

Treatment of 4T1 Tumors​: Tumors were induced by subcutaneous inoculation of 4T1 cells into the right flank. When tumor volumes attained approximately 100 mm^3^, mice were randomly allocated to two groups that received intravenous injections of either PBS or 4T1‐DTBEI@CS@MnO_2_ at three‐day intervals for a total of three or four administrations. 4T1‐DTBEI@CS@MnO_2_ was prepared according to the procedure described in the “*Preparation and characterization of DTBEI@CS@MnO_2_*” section, except that 4T1 cells were used instead of CT26 cells.

The same 4T1 cell line was used both for the preparation of DTBEI@CS@MnO_2_ and for the establishment of subcutaneous 4T1 tumor models. Mice were therefore divided into a Model (PBS) group and a 4T1‐DTBEI@CS@MnO_2_ treatment group. Tumor growth was monitored every 3 days, and tumor volume was calculated using the formula (width^2^ × length)/2.

At 24 h after the third injection, five mice from each group were sacrificed for the collection of tumor tissues and serum samples, while the remaining animals continued to be monitored for tumor growth. Tumor cell suspensions were prepared via grinding and filtration through a cell sieve. For DC detection, suspensions were stained with FITC‐anti‐mouse CD80 and PE‐anti‐mouse CD86. Concurrently, the remaining cell suspensions were stained with APC‐anti‐mouse CD3, FITC‐anti‐mouse CD4, and PE‐anti‐mouse CD8 for T cell profiling. Levels of cytokines including TNF‐α, IL‐6, IFN‐γ, and IL‐12 were quantified using respective ELISA kits.​

*Treatment of B16F10 Tumors*​: The identical protocol used for 4T1 tumors was applied to B16F10 tumors—establishment, B16F10‐DTBEI@CS@MnO_2_ therapy, and read‐outs—with 4T1 cells simply replaced by B16F10 cells.

### 4.27 Statistical Analysis

All quantitative data are expressed as mean ± SD. Statistical analyses were performed using GraphPad Prism 8. Differences between two groups were analyzed using a two‐tailed unpaired Student's *t*‐test. Differences among multiple groups were analyzed using one‐way ANOVA followed by Tukey's multiple‐comparisons test. Survival curves were generated using the Kaplan‐Meier method and compared using a two‐sided log‐rank (Mantel‐Cox) test. Animals remaining alive at the end of the predefined observation period were treated as censored observations. Statistical significance was defined as ^*^ *p* < 0.05, ^**^ *p* < 0.01, ^***^ *p* < 0.001, and ^****^ *p* < 0.0001, while “ns” denotes a non‐significant difference. Bar with color gradient was generated with Biodeep (https://www.biodeep.cn/home). Pathway enrichment of identified proteins was performed using DAVID (https://david.ncifcrf.gov/).

## Author Contributions

**Yiqun Wan**: conceptualization, methodology, funding acquisition, Writing – review and editing, project administration. **Bolun Xu**: methodology, writing – original draft, investigation, formal analysis. **Hao Wan**: conceptualization, methodology, formal analysis, investigation, funding acquisition, project administration, writing – review and editing. **Haihua Ji**: methodology. **Xuan Lu**: methodology. **Yuquan Wang**: methodology. **Ying Zhang**: project administration, writing – review and editing, funding acquisition, formal analysis, methodology.

## Funding

This work was financially supported by the National Natural Science Foundation of China (No. 32372322 and No. 82503553), the Central Guidance Fund for Local Science and Technology Development in Jiangxi Province (No. 20252ZDD020002), the Young Talent Cultivation Program of Nanchang University (No. XX202506030014), the Natural Science Foundation of Jiangxi Province (No. 20224ACB215009, No. 20232ACB205024, and No. 20242BAB21029), and the Research Project of State Key Laboratory of Food Science and Resources, Nanchang University (No. SKLF‐ZZB‐202529).

## Conflicts of Interest

The authors declare no conflicts of interest.

## Supporting information




**Supporting File**: advs77126‐sup‐0001‐SuppMat.docx.

## Data Availability

The data that supports the findings of this study are available in the supplementary material of this article.
