# Supplementary material for: Versatile Co‐Engineering of Immunogenic Cell Death‐Primed Dead Tumor Cell Bodies With Tumor‐Activated MnO2 for cGAS‐STING‐Potentiated Personalized Immunotherapy
Source: Adv Sci (Weinh). 2026 Aug 12;13(61):e77126. doi: 10.1002/advs.77126 (PMC13464454; doi:10.1002/advs.77126)
Supplement: Supplementary file 1 [file ADVS-13-e77126-s001.docx]

**Versatile co-engineering of immunogenic cell death-primed dead tumor cell bodies with tumor-activated MnO₂ for cGAS-STING-potentiated personalized immunotherapy**

*Bolun Xu, Xuan Lu, Yuquan Wang, Haihua Ji, Ying Zhang ^*^, Yiqun Wan ^*^, Hao Wan ^*^*

B. Xu, Y. Wan

School of Chemistry and Chemical Engineering, Nanchang University, Nanchang 330031, P. R. China.

E-mail: [wanyiqun@ncu.edu.cn](mailto:wanyiqun@ncu.edu.cn) (Y.W)

X. Lu, Y. Wang, H. Ji, Y. Zhang, Y. Wan, H. Wan

State Key Laboratory of Food Science and Resources, Nanchang University, Nanchang 330047, P. R. China.

E-mail: zhangying908@163.com (Y.Z.); wanhao424@ncu.edu.cn (H. W.)

**Figure S1.** Fluorescence images of CT26 cells or CT26-ICG stained with dihydroethidium (DHE) after 808 nm laser irradiation. Red fluorescence reflects intracellular ROS–induced oxidation of DHE to ethidium, followed by binding to cellular DNA or RNA. Scale bar, 150 μm.

**Figure S2.** Time-dependent temperature profiles of ICG or CT26-ICG solutions under 808 nm laser irradiation at a power density of 1 W/cm². Data are presented as mean ± S.D. (n = 3).

**Figure S3.** Viability of CT26 cells after internalization of ICG under laser irradiation (DTBEI) or lysis buffer treatment (LBTB). Data are presented as mean ± SD (n = 5). “ns” denotes no significant difference.

**Figure S4.** Extracellular ATP secretion from CT26 cells after different treatments. Data are presented as mean ± SD (n = 3). Statistical analyses were performed using one-way ANOVA; Significance is indicated as *****P* < 0.0001, and “ns” denotes no significance.

**Figure S5.** Quantification of CRT-associated extracellular signal in different treatment groups. Data are presented as mean ± SD (n = 3). Statistical analyses were performed using one-way ANOVA; Significance is indicated as *****P* < 0.0001, and “ns” denotes no significance.

**Figure S6.** HMGB1 release from CT26 cells after different treatments. Data are presented as mean ± SD (n = 3). Statistical analyses were performed using one-way ANOVA; Significance is indicated as ***P* < 0.01 or *****P* < 0.0001, and “ns” denotes no significance.

**Figure S7.** Proportion of mature DCs in splenocytes after stimulation with LBTB or DTBEI.

 **Figure S8.** Percentage of CD3⁺CD8⁺ T cells in splenocytes after stimulation with LBTB or DTBEI.

**Figure S9.** Changes in the proportion of CD3⁺CD4⁺ T-cell subset in splenocytes upon stimulation with LBTB or DTBEI.

**Figure S10.** Zeta potential shift of CT26-ICG cells after 15 min incubation with CS at 0-250 µg/mL. Data are expressed as mean ± SD (n = 3), with statistical significance denoted as *****P* < 0.0001.

**Figure S11.** Time-dependent zeta-potential change of CT26-ICG cells incubated with CS (200 µg/mL) for 0-20 min. Data are shown as mean ± SD (n = 3). The significance level is indicated as *****P* < 0.0001.

**Figure S12.** Representative X-Y optical section and Z-stack images of CT26-ICG cells incubated with Cy3-labeled CS under the optimized electrostatic assembly conditions. Red: Cy3; Green: DiO; Blue: DAPI.

**Figure** **S13.** Time-dependent zeta-potential change of CT26-ICG@CS incubated with 2 mM MnO₂ for 0-80 min. Data are shown as mean ± SD (n = 3). The significance level is denoted as *****P* < 0.0001.

**Figure S14.** Fluorescence intensity of FITC-labeled CS with different concentrations (i.e., 50, 100, 200, and 300 μg/mL)

**Figure S15.** Fluorescence calibration curve of FITC-labeled CS with different concentrations.

**Figure** **S16.** EDS analysis of DTBEI@CS@MnO_2_.

**Figure S17.** EDS line scan analysis of DTBEI@CS@MnO₂. **A)** SEM image with the yellow line indicating the EDS scan path across the cell surface. **B)** Corresponding EDS intensity profiles of C, Mn, O, P, and S as a function of scanning distance.

**Figure S18.** Stability of DTBEI@CS@MnO₂ in PBS over one week, characterized by zeta potential measurements (n = 3).

**Figure S19.** HMGB1 leakage and TME-triggered release profiles of different DTBEI-

based formulations. Data are presented as mean ± SD (n = 3).

**Figure S20.** Representative flow cytometry histograms showing antibody-accessible CRT in untreated CT26 cells, CT26-ICG cells without laser, free DTBEI, DTBEI@CS, DTBEI@CS@MnO₂ incubated under pH 7.4 conditions, and DTBEI@CS@MnO₂ incubated under pH 6.5 plus GSH conditions.

**Figure S21.** Rank-abundance analysis of membrane-associated proteins in DTBEI and DTBEI@CS@MnO₂ before and after storage at 37 °C. The abundance of each membrane-associated protein was normalized to that in fresh DTBEI at day 0, which served as the common baseline reference.

**Figure S22.** Heatmap analysis of representative membrane-associated and cell-surface proteins in DTBEI and DTBEI@CS@MnO₂ before and after storage at 37 °C.

**Figure S23.** Time-course SDS-PAGE analysis of total-protein profiles in DTBEI and DTBEI@CS@MnO₂ during storage at 37 °C.

**Figure S24.** Quantification of the background-subtracted total-lane intensity normalized to the corresponding fresh day-0 sample. Data are presented as mean ± SD (n = 3). Statistical analysis was performed using Student’s *t*-test. ^****^*P* < 0.0001.

**Figure S25.** Representative Western blot images of HMGB1, HSP90, EpCAM, and integrin β1 in fresh samples and samples stored for seven days. 1: DTBEI D0; 2: DTBEI D7; 3: DTBEI@CS@MnO_2_ D0; 4: DTBEI@CS@MnO_2_ D7.

**Figure S26.** Densitometric quantification of HMGB1, integrin β1, HSP90, and EpCAM normalized to the corresponding GAPDH signals. Data are presented as mean ± SD (n = 3). Statistical significance was assessed by one-way ANOVA. ^*^*P* < 0.05, ^***^*P* < 0.001, and ^****^*P* < 0.0001; ns, not significant.

**Figure S27.** IFN-γ production in mouse splenocytes after stimulation with different formulations under tumor-mimicking (pH 6.5) and physiological (pH 7.4) conditions. Data are presented as mean ± SD (n = 3). Statistical analysis was performed using one-way ANOVA. ns, not significant; ^*^*P* < 0.05, ^**^*P* < 0.01, and ^***^*P* < 0.001.

**Figure S28.** *In vitro* serum stability assay of DiD-labeled DTBEI@CS@MnO₂, showing the DiD leakage ratio over 72 h. Data are expressed as mean ± SD (n = 3).

**Figure S29.** *In vivo* fluorescence imaging of CT26 tumor-bearing mice after intravenous injection of PBS, free DiD, or DiD-labeled DTBEI@CS@MnO₂ at different time points (i.e., 0, 6, 10, 48, and 72 h). The white dashed circles indicate tumor regions.

**Figure S30.** *Ex vivo* fluorescence imaging of tumors, major organs (i.e., heart, liver, spleen, lung, kidney), and lymph node collected at 10 h post injection.

**Figure S31.** Schematic of tumor treatment protocols based on different injection numbers.

**Figure S32.** Tumor volume changes over time in CT26 tumor-bearing mice treated with DTBEI@CS@MnO₂ for different times.

**Figure S33.** Time-dependent changes in tumor volume of mice following different treatments. Data are presented as mean ± SD (n = 5). One-way ANOVA was employed for statistical analysis, with a significance level of ^****^*P* < 0.0001.

**Figure S34.** Comparative size of resected tumors after different treatments. Data are presented as mean ± SD (n = 5). One-way ANOVA was used for statistical analysis, with significance indicated as ^****^*P* < 0.0001.

**Figure S35.** Tumor volume changes in CT26 tumor-bearing mice after treatment with PBS, MnO₂, DTBEI, DTBEI@CS, DTBEI+CS+MnO₂, or DTBEI@CS@MnO₂.

**Figure S36.** Representative images of excised CT26 tumor tissues from mice treated with different formulations at the end of the *in vivo* study.

**Figure S37.** Kaplan-Meier survival analysis of CT26 tumor-bearing mice treated with PBS or DTBEI@CS@MnO₂. Survival curves were compared using a two-sided log-rank (Mantel-Cox) test (n = 8; ^****^*P* < 0.0001).

**Figure S38.** Serum levels of inflammatory cytokines (i.e., IFN-β, IL-6, and TNF-α) in normal mice after treatment with PBS, MnO₂ and DTBEI@CS@MnO₂. Data are expressed as mean ± SD (n = 3). One-way ANOVA was used for statistical analysis, with no significance indicated as “ns”.

**Figure S39.** WB analysis of the STING pathway activation in liver tissues from mice treated with PBS, MnO₂, or DTBEI@CS@MnO₂. A) Representative western blot bands of p-STING, p-TBK1, and p-IRF3. GAPDH was used as the loading control. B) Quantitative analysis of the relative gray intensity of p-STING, p-TBK1, and p-IRF3, normalized to the corresponding GAPDH levels. Data are expressed as mean ± SD (n = 3). One-way ANOVA was used for statistical analysis, with no significance indicated as “ns”.

**Figure S40.** WB analysis of the STING pathway activation in spleen tissues from mice treated with PBS, MnO₂, or DTBEI@CS@MnO₂. A) Representative western blot bands of p-STING, p-TBK1, and p-IRF3. GAPDH was used as the loading control. B) Quantitative analysis of the relative gray intensity of p-STING, p-TBK1, and p-IRF3, normalized to the corresponding GAPDH levels. Data are expressed as mean ± SD (n = 3). One-way ANOVA was used for statistical analysis, with no significance indicated as “ns”.

**Figure S41.** WB analysis of the STING pathway activation in lung tissues from mice treated with PBS, MnO₂, or DTBEI@CS@MnO₂. A) Representative western blot bands of p-STING, p-TBK1, and p-IRF3. GAPDH was used as the loading control. B) Quantitative analysis of the relative gray intensity of p-STING, p-TBK1, and p-IRF3, normalized to the corresponding GAPDH levels. Data are expressed as mean ± SD (n = 3). One-way ANOVA was used for statistical analysis, with no significance indicated as “ns”.

**Figure** **S42.** Serum biochemical parameters including ALB, ALT, AST, TG, UA, CK, and TBA in normal mice after intravenous injection of PBS, MnO₂, and DTBEI@CS@MnO₂. The values indicated at the top of each graph represent the corresponding physiological reference intervals provided by the laboratory performing the biochemical measurements using the same analytical platform. These reference intervals were used to assess potential deviations from normal physiological levels. Data are presented as mean ± SD (n = 3). Statistical differences were analyzed via one-way ANOVA, and non-significant results were marked as “ns”.

**Figure S43.** Representative H&E-stained sections of major organs, including heart, liver, spleen, lung, and kidney, collected from normal mice after treatment with PBS, MnO₂ and DTBEI@CS@MnO₂. Scale bar = 50 μm.

**Figure S44.** Intratumoral IFN-γ levels in CT26 tumors following treatment with different formulations. Data are presented as mean ± SD (n = 3). Statistical analysis was performed using one-way ANOVA. ns, not significant; ^****^*P* < 0.0001.

**Figure S45.** Quantification of splenic CD44⁺CD62L⁺ double-positive cells following homologous CT26 rechallenge. Data are presented as mean ± SD (n = 3). Statistical significance was evaluated using Student’s *t*-test. ^****^*P* < 0.0001.

**Figure S46.** Zeta-potential variations accompanying sequential coating of 4T1-DTBEI@CS@MnO₂ (n = 3).

**Figure S47.** Zeta-potential signatures for each intermediate in B16F10-DTBEI@CS@MnO₂ synthesis (n = 3).

**Figure S48.** SEM micrographs comparing surface topography of native 4T1 cell and 4T1-DTBEI@CS@MnO₂. Scale bar, 5 μm.

**Figure S49.** Surface topography of native B16F10 cell and B16F10-DTBEI@CS@MnO₂ observed by SEM. Scale bar, 5 μm.

**Figure S50.** Time-dependent 4T1 tumor volume in mice following 4T1-DTBEI@CS@MnO₂ treatment.

**Figure S51.** Time-dependent changes in B16F10 tumor volume in mice following B16F10-DTBEI@CS@MnO₂ treatment (n = 5).

**Figure S52.** Time-dependent growth curves of B16F10 tumor in each mouse after B16F10-DTBEI@CS@MnO₂ treatment.

**Figure S53.** *Ex vivo* digital photo of B16F10 tumors harvested post-treatment. 1: Model; 2: B16F10-DTBEI@CS@MnO_2_.

**Figure** **S54.** Resected B16F10 tumor sizes post-treatment. Data are shown as mean ± SD (n = 5). Statistical analysis was performed by Student’s *t*-test; significance: ^****^*P* < 0.0001.

**Figure S55.** Flow-cytometric quantification of DC maturation (CD80^+^CD86^+^) in B16F10 tumors from various groups. Q2 quadrant indicates percentage of mature DC across different groups.

**Figure S56.** Flow-cytometry analysis showing CD3^+^CD4^+^ T-cell abundance in B16F10 tumors resected from B16F10-DTBEI@CS@MnO₂-treated mice. The upper right quadrant represents CD3⁺CD4⁺ T-cell proportion.

**Figure S57.** Flow-cytometric analysis of CD3^+^CD8^+^ T-cell surface markers in B16F10 tumors. The upper right quadrant indicates the percentage of CD3⁺CD8⁺ cytotoxic T cells from mice treated with B16F10-DTBEI@CS@MnO₂.

**Figure S58.** Intratumoral IFN-γ levels in 4T1 tumors after treatment with DTBEI@CS@MnO₂. Data are presented as mean ± SD (n = 3). ns, not significant; ^****^*P* < 0.0001.

**Figure S59.** Cytokine (i. e., TNF-α, IL-6, IFN-γ, and IL-12) levels in B16F10 tumor-bearing mice following B16F10-DTBEI@CS@MnO₂ treatment. 1: Model; 2: B16F10-DTBEI@CS@MnO₂. Data are shown as mean ± SD (n = 3). Statistical significance was assessed via Student’s *t*-test; ^****^*P* < 0.0001 corresponds to a significant difference.

**Figure S60.** Representative immunofluorescence images showing M1 macrophage marker iNOS (Cy3, red) and M2 macrophage marker CD206 (FITC, green) in B16F10 tumors from the Model and B16F10-DTBEI@CS@MnO₂ groups. Cell nuclei were counterstained with DAPI (blue). Scale bar, 50 μm.

**Figure S61.** Representative immunofluorescence images showing Foxp3⁺CD4⁺ regulatory T cells in B16F10 tumors from the Model and B16F10-DTBEI@CS@MnO₂ groups. Foxp3 was stained with SpGreen-labeled recombinant anti-Foxp3 antibody (green) and CD4 was stained with SpOrange-labeled recombinant anti-CD4 antibody (red). Cell nuclei were counterstained with DAPI (blue). Scale bar, 50 μm.
